# Identification and functional validation of EPS15L1 as a key driver of triple-negative breast cancer

**DOI:** 10.1038/s41523-026-00954-9

**Published:** 2026-05-15

**Authors:** Chun-Chao Wang, Sabareeswaran Krishnan, Yen-Hsun Wang, Kai-Chen Hsu, Tzu-Sheng Hsu, Chung-Hsing Chen, Shang-Hung Chen, Ren-Hua Chung

**Affiliations:** 1 Institute of Molecular Medicine, National Tsing Hua University, Hsinchu, Taiwan; 2 Interdisciplinary Program of Life Sciences and Medicine, National Tsing Hua University, Hsinchu, Taiwan; 3 Institute of Molecular and Cellular Biology, National Tsing Hua University, Hsinchu, Taiwan; 4 Department of Mathematics, University of Taipei, Taipei, Taiwan; 5 National Institute of Cancer Research, National Health Research Institutes, Zhunan, Taiwan; 6 National Institute of Cancer Research, National Health Research Institutes, Tainan, Taiwan; 7 Department of Oncology, National Cheng Kung University Hospital, College of Medicine, National Cheng Kung University, Tainan, Taiwan; 8 Institute of Population Health Sciences, National Health Research Institutes, Zhunan, Taiwan

**Keywords:** Biomarkers, Cancer, Computational biology and bioinformatics, Oncology

## Abstract

Triple-negative breast cancer (TNBC) is a heterogeneous and aggressive subtype with no effective targeted therapies. To identify novel therapeutic targets, we conducted transcriptome-wide association studies (TWAS) integrating genome-wide association studies (GWAS) summary statistics from the Breast Cancer Association Consortium (BCAC) and UK Biobank with expression quantitative trait loci (eQTL) summary statistics from breast mammary tissues via the Genotype-Tissue Expression (GTEx) study. Our approach identified novel candidate genes positively or negatively associated with TNBC based on their TWAS effect size. We validated these candidates by confirming their differential expression between TNBC and normal tissues using The Cancer Genome Atlas (TCGA) breast cancer cohort. Among these, Epidermal Growth Factor Receptor Pathway Substrate 15 Like 1 (EPS15L1) emerged as a top candidate significantly associated with TNBC. EPS15L1 was found to be overexpressed in basal-like and claudin-low TNBC tumors and cell lines, with its high expression correlating with poor prognosis. Functional studies demonstrated that EPS15L1 enhances TNBC cell growth, as evidenced by increased metabolic activity in high-density conditions, enhanced clonogenic ability in low-density settings, and improved 3D spheroid formation. Additionally, knockdown of EPS15L1 suppressed the formation and stellate morphology of single-cell-initiated spheroids within the 3D microenvironment. Collectively, these findings establish EPS15L1 as a driver of TNBC initiation, implying its potential as a biomarker for early detection and a target for prevention.

## Introduction

Triple-negative breast cancer (TNBC) is clinically defined by negative immunohistochemical expression of estrogen receptor (ER) and progesterone receptor (PR) (typically <1% nuclear staining)^1^ and by HER2 negativity, defined as HER2 immunohistochemistry (IHC) score 0/1+ or IHC 2+ without ERBB2 amplification by in situ hybridization (ISH)^2^. TNBC accounts for ~15–20% of breast cancers and is associated with higher rates of early relapse and metastasis than other major subtypes, leaving substantial unmet needs despite an expanding treatment landscape that now includes immunotherapy and antibody–drug conjugates (ADCs) for selected clinical settings^3–6^. Beyond its clinical definition, TNBC is biologically heterogeneous: transcriptomic and microenvironmental profiling has resolved TNBC into multiple molecular states with distinct signaling dependencies and therapeutic sensitivities, including basal-like programs and mesenchymal/claudin-low–like phenotypes^7,8^, as well as refined subtype frameworks such as basal-like 1 (BL1), basal-like 2 (BL2), immunomodulatory (IM), mesenchymal (M), mesenchymal stem-like (MSL), luminal androgen receptor (LAR), and unclassified subtypes^9–12^. This heterogeneity complicates target discovery and contributes to variable clinical behavior and treatment response.

At the genomic level, TNBC tumors harbor recurrent somatic alterations, yet actionable targets remain limited for most patients. Large-scale tumor sequencing efforts (e.g., TCGA) have highlighted frequent driver events such as TP53 alterations, but relatively few therapeutically tractable dependencies have translated into broadly effective targeted therapies^13,14^. Germline susceptibility is also clinically relevant: pathogenic variants in BRCA1/2 and other DNA repair genes occur in a meaningful subset of TNBC and influence treatment decisions^15^, underscoring the importance of defining inherited mechanisms that predispose to TNBC and may reveal new vulnerabilities. Consistent with this need, genome-wide association studies (GWAS) have mapped hundreds of breast cancer susceptibility loci, including loci that show stronger associations with ER-negative/TNBC disease^16,17^. Among these, the 19p13.1 region is an early TNBC/ER-negative–enriched susceptibility locus, initially identified as a BRCA1 modifier and subsequently supported by large subtype-focused analyses^18–22^. However, as with many GWAS loci, pinpointing causal genes and mechanisms remains challenging because of linkage disequilibrium, regulatory pleiotropy, and context-dependent gene regulation.

Transcriptome-wide association studies (TWAS) provide a complementary strategy to connect GWAS signals to candidate genes by leveraging expression quantitative trait loci (eQTLs) to infer genetically regulated expression and test its association with disease risk^23–28^. Large breast cancer TWAS efforts have nominated numerous candidate susceptibility genes and helped prioritize regulatory mechanisms not apparent from variant-level associations^29–31^. Nonetheless, TWAS associations can reflect correlated regulation or linkage among eQTLs^32,33^, and recent methodological work highlights the need for careful interpretation using approaches such as colocalization and fine-mapping^34^, alongside strategies that mitigate genetic confounding and, critically, experimental validation to establish functional relevance.

In parallel, contemporary TNBC biology has reinforced the centrality of receptor tyrosine kinase (RTK) signaling—particularly EGFR pathway activity in basal-like contexts^35,36^—yet clinical trials targeting EGFR in TNBC have generally shown limited benefit^37–39^, motivating alternative strategies to modulate pathway output. Endocytosis and membrane trafficking are now recognized as key determinants of RTK signaling amplitude, duration, and spatial compartmentalization, thereby shaping downstream phenotypes relevant to tumor progression. Clathrin-mediated endocytosis (CME) can sustain or reprogram EGFR signaling by controlling receptor internalization, recycling, and degradation, and dysregulated endocytic networks are increasingly implicated in oncogenic signaling and therapeutic resistance^40^. EPS15L1 (epidermal growth factor receptor pathway substrate 15-like 1), a component of clathrin-coated pits involved in receptor internalization, is therefore a mechanistically plausible link between inherited regulatory variation and altered EGFR network behavior^41–43^; however, its role in TNBC pathogenesis has remained insufficiently defined.

Based on this background, we hypothesized that TWAS integrating breast tissue eQTLs with TNBC GWAS data would identify genes whose genetically regulated expression is specifically associated with TNBC risk, and that EPS15L1, as a regulator of EGFR endocytosis, promotes TNBC progression. Our objectives were: (1) to identify novel TNBC susceptibility genes using TWAS; (2) to validate candidate genes in independent expression datasets; (3) to functionally characterize the role of the top candidate, EPS15L1, in TNBC cell growth and tumor-initiating capacity; and (4) to assess the prognostic significance of EPS15L1 expression in TNBC patients.

Here, we performed TWAS for overall breast cancer and TNBC using GWAS summary statistics from the Breast Cancer Association Consortium (BCAC) and UK Biobank, integrating breast tissue eQTL resources to identify TNBC susceptibility genes in European-ancestry cohorts. We identified distinct sets of putative TNBC suppressor and promoter genes, highlighting EPS15L1 as a prominent TNBC promoter gene with the strongest association signal for TNBC and a more modest association with overall breast cancer. These findings were supported by gene expression analyses in the TCGA breast cancer cohort. Using a panel of TNBC cell lines, we further show that EPS15L1 is frequently overexpressed in TNBC and promotes TNBC cell growth, particularly under low-density 2D culture conditions. Moreover, EPS15L1 is required for single-cell–initiated spheroid formation and stellate morphology in 3D cultures, and higher EPS15L1 expression correlates with poorer prognosis in TNBC patients. Collectively, our work prioritizes novel TNBC susceptibility genes from human genetics and establishes EPS15L1 as a candidate biomarker and therapeutic target linking inherited regulatory architecture to endocytic control of growth factor signaling in TNBC.

## Results

### Study overview and key findings

**Fig. 1:**
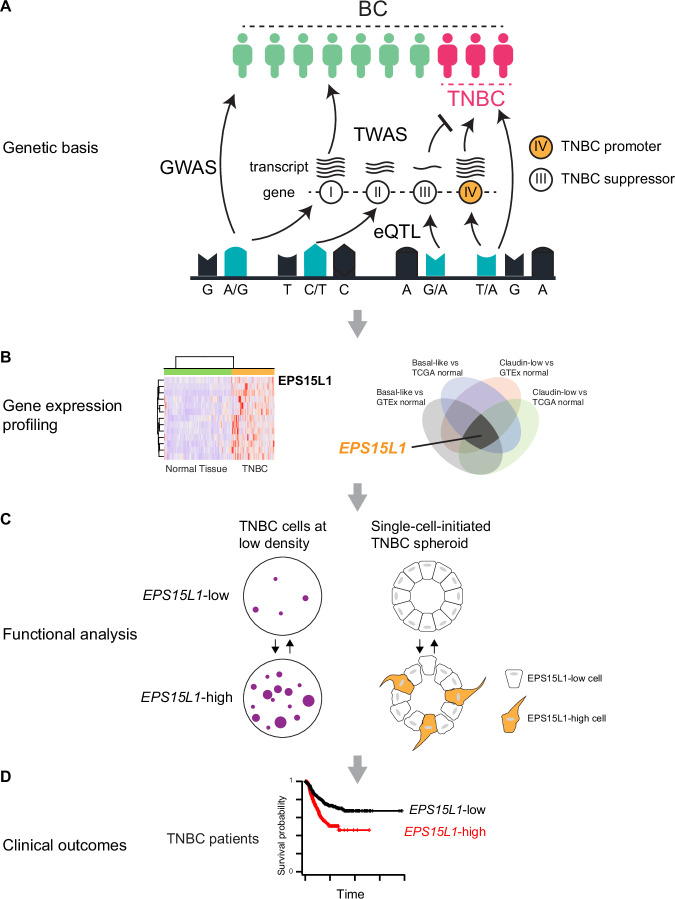
Study overview and key findings. **A** Schematic of the integrative GWAS–TWAS framework used to prioritize candidate genes associated with breast cancer (BC) and triple-negative breast cancer (TNBC). GWAS identifies trait-associated variants, and TWAS tests associations between genetically predicted gene expression (from breast eQTL-based prediction models) and disease risk to nominate candidate genes. **B** Gene-expression profiling comparing normal breast tissue versus TNBC tumors and identifying genes consistently altered across TNBC subtypes (basal-like and claudin-low) in TCGA/GTEx comparisons; EPS15L1 is highlighted as a prioritized candidate. **C** Overview of functional validation assays used in cellular models, testing the impact of EPS15L1-low vs EPS15L1-high states on TNBC growth under low-density 2D conditions and single-cell–initiated 3D spheroid formation. **D** Clinical relevance assessed by survival analysis comparing TNBC patient groups with high vs low EPS15L1 expression.

**Fig. 2:**
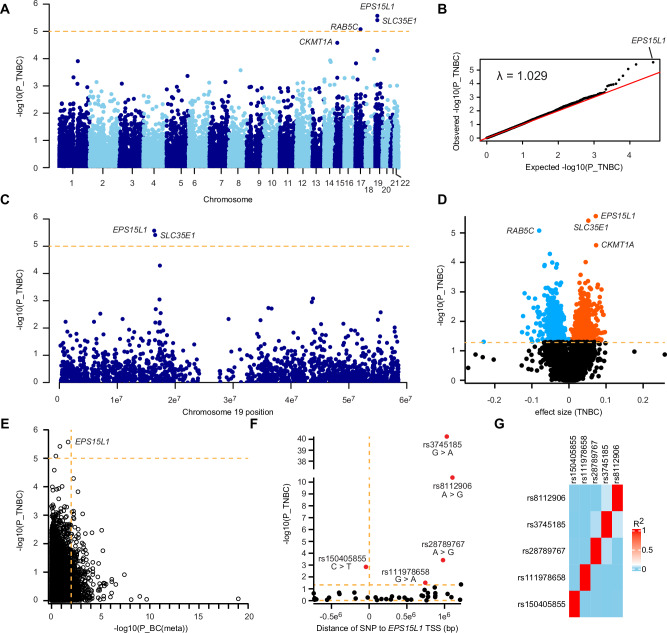
TWAS association mapping identifies candidate TNBC genes and EPS15L1-associated eQTLs. **A** Manhattan plot of TWAS results for TNBC risk. The x-axis shows chromosomal position and the y-axis shows −log10(P_TNBC). The horizontal dashed line denotes the prespecified TWAS significance threshold (*P* = 1 × 10⁻⁵). Each point represents a gene or non-coding transcript; selected top signals are labeled. **B** Quantile–quantile (Q–Q) plot of TWAS P values for TNBC; the genomic inflation factor (λ) is shown. **C** Regional TWAS association plot across chromosome 19 (x-axis: genomic position; y-axis: −log10(P_TNBC)). **D** Volcano plot summarizing TWAS associations with TNBC. The x-axis shows TWAS effect size and the *y*-axis shows −log10(P_TNBC). Genes meeting nominal significance (*P* < 0.05) are colored by effect direction (positive effect, orange; negative effect, blue); non-significant genes are shown in black. Labeled genes indicate the top four associations. **E** Scatter plot comparing TWAS significance for overall breast cancer versus TNBC (x-axis: −log10(P_BC(meta)); y-axis: −log10(P_TNBC)). The vertical dashed line indicates −log10(P_BC(meta)) = 2 (P_BC(meta) = 0.01), and the horizontal dashed line indicates −log10(P_TNBC) = 5 (P_TNBC = 1 × 10⁻⁵). **F** Association of EPS15L1 eQTLs with TNBC risk plotted by distance to the EPS15L1 transcription start site (TSS) (x-axis: distance to TSS in bp; y-axis: −log10(P_TNBC)). The five EPS15L1 eQTLs in the prediction model (rs150405855, rs111978658, rs28789767, rs3745185, rs8112906) are annotated; the y-axis is shown with a break to display highly significant variants. **G** Pairwise linkage disequilibrium (LD; r²) among the five EPS15L1 eQTLs, showing low LD and suggesting largely independent contributions to TNBC risk.

Figure 1 outlines our analytical workflow. Initially, gene expression prediction models were developed using eQTL summary statistics from breast mammary tissues, sourced from the GTEx study samples^27^ and accessed via the eQTL Catalog^44^. Subsequently, we carried out a TWAS for TNBC, integrating these models with GWAS summary statistics from the Breast Cancer Association Consortium (BCAC)^16^, employing the S-PrediXcan method (Fig. 2)^28^. Additionally, we performed two separate TWAS for overall breast cancer (BC). The first relied on GWAS summary statistics from the BCAC, employing S-PrediXcan (Fig. S1), while the second used data from UK Biobank samples, applying PrediXcan (Fig. S2)^24^. A meta-analysis was then applied to combine these two sets of TWAS findings (Fig. S3).

The expression of genes strongly associated with TNBC was compared between TNBC and normal breast tissue using data from the TCGA, GTEx, and various cell lines (Fig. 1B). This analysis identified genes that were highly correlated with both basal-like and claudin-low TNBC subtypes compared to controls, with a particular emphasis on *EPS15L1*. To validate our findings experimentally in vitro, *EPS15L1* expression was manipulated in both non-tumorigenic and TNBC cells (Fig. 1C). We then compared cell viability, colony formation ability, and single-cell-initiated spheroid formation ability between these manipulated cells. Furthermore, to gauge the clinical relevance of EPS15L1 in TNBC, we conducted a patient survival analysis (Fig. 1D), which underscores the potential of EPS15L1 as a prognostic marker in TNBC treatment strategies.

### Genome- and transcriptome-wide association studies

The gene-based meta-analysis resulted in several genes being genome-wide significantly associated with TNBC on chromosomes 17 and 19, based on the *p*-values for TNBC (Fig. 2A–D, Figs. S1–3, Table 1, and Data S1). The top genes included *EPS15L1* (*P* = 2.69 × 10^−6^), *SLC35E1* (*P* = 3.84 × 10^−6^), and *RAB5C* (*P* = 8.36 × 10^−6^). A comparison of *P*-values for TNBC and overall breast cancer revealed low concordance between the two datasets, likely reflecting the distinct genetic basis of TNBC and the unique contribution of germline variants to TNBC susceptibility (Fig. 2E). We then focused further analysis on the epidermal growth factor receptor pathway substrate 15 like 1 (EPS15L1), the gene with the most significant association with TNBC, which also showed a modest association with overall breast cancer risk (Fig. 2A–E).

**Table 1:** The association statistics of the SNPs with *EPS15L1* expression, TNBC, and overall BC

eQTL	chromosome	Position	Gene expression^a^	TNBC^b^	Overall BC (BCAC)^c^	Overall BC (UKB)^d^
rs150405855	19	16313122	0.622 1.78E-02	0.394 (0.123) 1.42E-03	-0.008 (0.035) 8.05E-01	0.042 (0.059) 4.77E-01
rs111978658	19	17099674	-0.528 1.27E-02	0.087 (0.040) 3.07E-02	0.023 (0.022) 2.84E-01	-0.061 (0.038) 1.11E-01
rs28789767	19	17334178	0.213 1.20E-04	0.043 (0.012) 3.82E-04	0.002 (0.006) 6.87E-01	-0.009 (0.013) 5.07E-01
rs3745185	19	17384267	-0.137 1.48E-02	-0.159 (0.012) 5.63E-41	-0.031 (0.006) 1.79E-07	-0.011 (0.013) 4.11E-01
rs8112906	19	17459147	-0.221 5.30E-04	-0.098 (0.014) 4.13E-11	-0.012 (0.007) 1.09E-01	-0.018 (0.015) 2.42E-01

^a^The beta estimate and *p*-value of the eQTL from the joint prediction model for *EPS15L1* gene expression

^b^The association statistics (beta, se, *p*-value) of the eQTL with TNBC based on the BCAC data

^c^The association statistics (beta, se, *p*-value) of the eQTL with overall BC based on the BCAC data

^d^The association statistics (beta, se, *p*-value) of the eQTL with overall BC based on the UK Biobank (UKB) data

Table 1 provides detailed summary statistics for the eQTLs for the EPS15L1 gene, focusing specifically on those eQTLs with association *p*-values for TNBC < 0.05 (Fig. 2F). A complete list of association statistics pertaining to the EPS15L1 eQTLs can be found in Data S2. Notably, the 5 eQTLs highlighted in Table 1 were characterized by low pairwise linkage disequilibrium (LD) (Fig. 2G), indicating that they may have independent effects on TNBC. The pairwise LD *r*^*2*^ of these eQTLs are provided in Data S3. The two eQTLs rs3745185 and rs8112906 showed genome-wide significant *p*-values for TNBC. The remaining three eQTLs, while not as prominent, also demonstrated some degree of association with TNBC.

### Specificity of *EPS15L1* association at the 19p13.11 susceptibility locus

In our TWAS, *EPS15L1* and the neighboring *SLC35E1* ( ~ 350 kb apart at 19p13.11) were both among the top TNBC-associated genes (Fig. 2A and 2C). To determine whether the cis-eQTLs underlying the *EPS15L1* signal might also regulate *SLC35E1*, we queried the five cis-eQTLs retained in our joint *EPS15L1* prediction model (rs150405855, rs111978658, rs28789767, rs3745185, rs8112906; Table 1). These variants were selected from GTEx breast mammary tissue eQTL resources (via the eQTL Catalog) and exhibit low pairwise LD (Fig. 2G), supporting multiple independent regulatory components within the locus. Notably, none of these *EPS15L1*-model variants showed evidence of being *SLC35E1* eQTLs in our lookup, indicating that the genetically regulated component captured for *EPS15L1* is not explained by shared regulation of *SLC35E1*.

Consistent with *SLC35E1* being a nearby TWAS hit yet potentially representing an independent signal, *SLC35E1* expression showed subtype-dependent elevation: it was significantly higher in basal-like tumors compared with GTEx normal (*P* = 7.65 × 10⁻³¹; means 4446.57 vs 1705.27) and TNBC-adjacent normal (*P* = 4.13 × 10⁻⁷; means 4446.57 vs 3414.17), but not different between claudin-low tumors and TNBC-adjacent normal (*P* = 0.733; means 3345.56 vs 3414.17) (Fig. S4).

Finally, these findings are placed in the context of prior genetics: 19p13.1/19p13.11 is a well-established susceptibility region for ER-negative/TNBC^18–22^, initially identified as a BRCA1-modifier locus and subsequently validated as TNBC-enriched in large subtype-focused analyses and follow-up fine-mapping/functional work.

### Cross-ancestry validation

To evaluate the relevance of our findings beyond European ancestry populations, we conducted cross-ancestry validation using GWAS summary statistics for TNBC in women of African ancestry (2,860 cases and 16,262 controls) reported by Jia et al.^29^. Among the two EPS15L1 eQTLs that were genome-wide significant in European cohorts, rs3745185 remained significant (*P* = 1.59 × 10⁻⁷), while rs8112906 showed marginal significance (*P* = 0.057). However, TWAS of EPS15L1 based on the African ancestry dataset did not yield significant results (*P* = 0.821). This discrepancy may reflect limited overlap in predictive variants, as only 26 of the 41 eQTLs used in our expression model were present in the African dataset, as well as differences in effect directions for several of these overlapping eQTLs. These findings provide partial cross-ancestry support for *EPS15L1* while underscoring the importance of incorporating diverse populations to fully elucidate its role in TNBC susceptibility.

### Tumor suppressor genes differentially expressed in basal-like and claudin-low TNBC

**Fig. 3:**
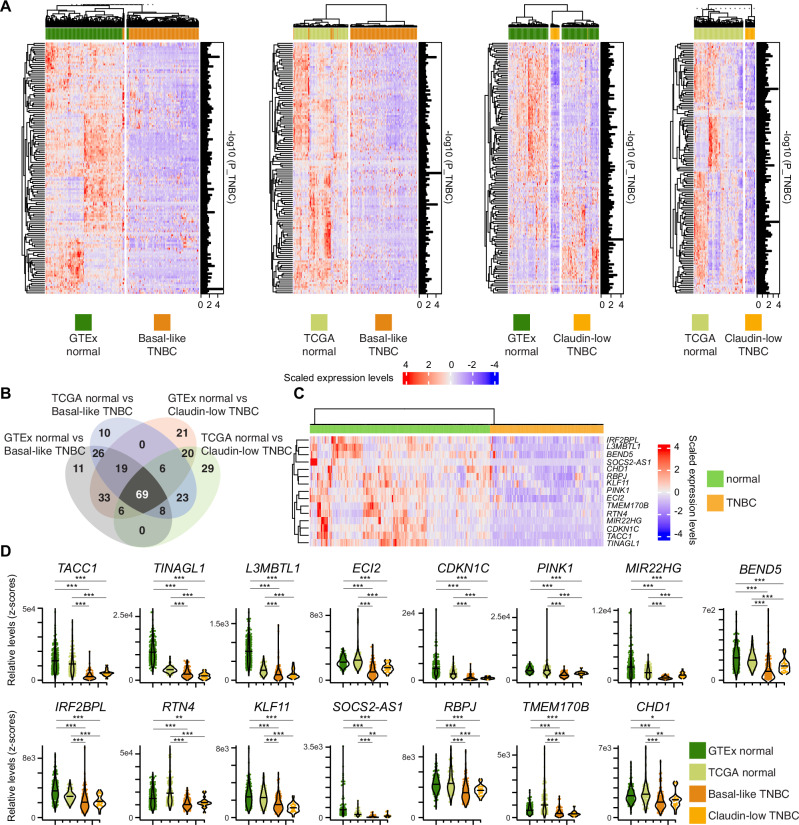
Differential expression of anti-TNBC genes distinguishes normal breast tissue from TNBC. **A** Heatmaps of anti-TNBC genes differentially expressed in basal-like and claudin-low TNBC compared with normal breast tissue using the GTEx and TCGA datasets. Expression values are shown as scaled (z-score) expression. The number of differentially expressed genes for each comparison is: GTEx normal vs basal-like TNBC (*n* = 131), TCGA normal vs basal-like TNBC (*n* = 128), GTEx normal vs claudin-low TNBC (*n* = 141), and TCGA normal vs claudin-low TNBC (*n* = 138). **B** Venn diagram showing the overlap of differentially expressed genes across the four comparisons. **C** Heatmap of top candidate tumor suppressor genes downregulated in TNBC compared with normal tissue. **D** Expression of the indicated tumor suppressor genes in GTEx normal breast tissue (*n* = 179), TCGA normal breast tissue (*n* = 113), TCGA basal-like TNBC (*n* = 162), and TCGA claudin-low TNBC (*n* = 22). The black bar denotes the mean. Significance: *FDR < 0.05, **FDR < 0.01, ***FDR < 0.001 (Benjamini–Hochberg FDR–adjusted *P* values; Welch’s two-sided *t*-test).

**Fig. 4:**
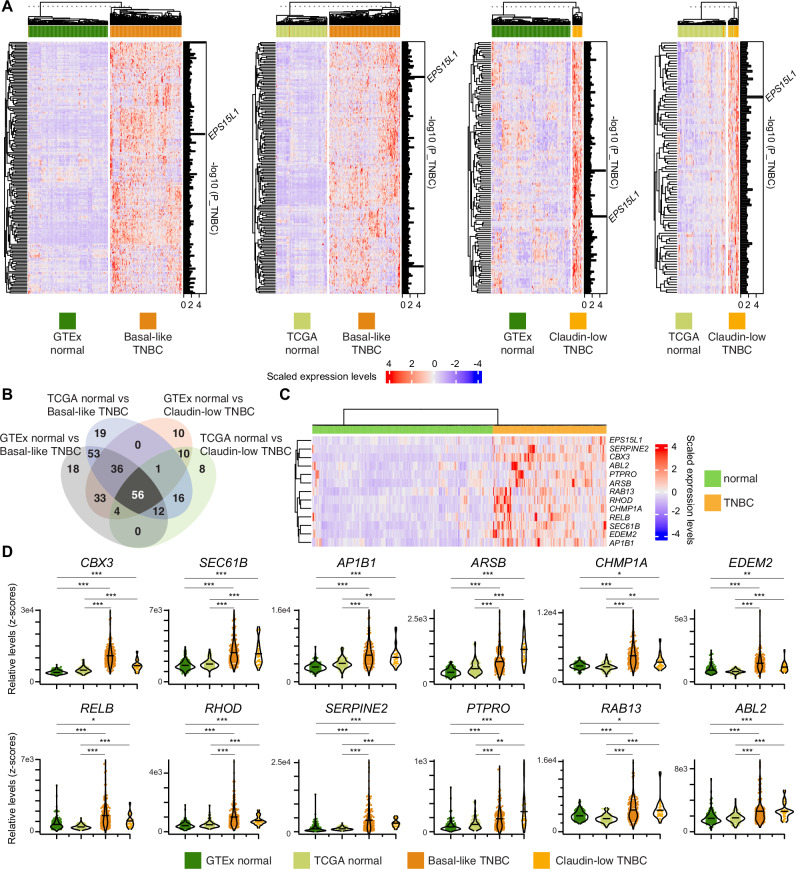
Pro-TNBC genes differentially upregulated in TNBC subtypes compared to normal tissue. **A** Heatmaps show pro-TNBC genes differentially expressed in basal-like and claudin-low TNBC relative to normal breast tissue using GTEx and TCGA datasets, with hierarchical clustering of genes by expression pattern. Numbers of differentially expressed genes are: GTEx normal vs basal-like TNBC (*n* = 176), TCGA normal vs basal-like TNBC (*n* = 161), GTEx normal vs claudin-low TNBC (*n* = 132), and TCGA normal vs claudin-low TNBC (*n* = 98). **B** Venn diagram shows overlap of differentially expressed pro-TNBC genes across the four comparisons. **C** Heatmap shows top candidate tumor-promoter genes (EGFR endocytosis/signaling–related) upregulated in TNBC relative to normal tissues. **D** Expression (z-score) of indicated tumor-promoter genes in GTEx normal breast tissue (*n* = 179), TCGA solid normal breast tissue (*n* = 113), TCGA basal-like TNBC (*n* = 162), and TCGA claudin-low TNBC (*n* = 22). The black bar denotes the mean. Significance: *FDR < 0.05, **FDR < 0.01, ***FDR < 0.001 (Benjamini–Hochberg FDR–adjusted *P* values; Welch’s two-sided t-test).

In a transcriptomic analysis of a TCGA-BRCA cohort of primary TNBC tumors and solid normal breast epithelial tissue from TCGA and GTEx^45,46^, we focused on genes with *p* < 0.05 for TNBC, and either positive or negative effect sizes for TNBC (Figs. 2D, 3A, and 4A). Following manual curation and review of detectable, differentially expressed TNBC-related genes with negative effect sizes for TNBC across four sample groups (GTEx normal tissue, TCGA normal tissue, basal-like TNBC, and claudin-low TNBC), we identified 69 potential tumor suppressor genes (Fig. 3B and S5). These genes were downregulated in both basal-like and claudin-low TNBC subtypes compared to normal tissues (GTEx and TCGA) (Fig. 3A–D). Functional annotations indicate that these genes play roles in regulating cell division, signal transduction pathways, and tumor suppressive functions. They impact therapeutic outcomes and prognosis, and inhibit cancer growth and metastasis in various cancers including TNBC, lung, colorectal, gastric, and breast cancers. Specific examples include genes involved in EGFR inhibition, triggering of cell death signaling, enhancement of targeted therapy, disruption of cancer cell division, and suppression of TNBC, endometrial and colorectal cancer progression. These genes include *TACC1*^47^, *TINAGL1*^48^, *L3MBTL1*^49^, *ECI2*^50^, *CDKN1C*^51^, *PINK1*^52^, *MIR22HG*^53^, *BEND5*^54^, *IRF2BPL*^55^, *RTN4*^56^, *KLF11*^57^, *SOCS2-AS1*^58^, *RBPJ*^54^, *TMEM170B*^59^, and *CHD1*^60^ (Table S1). Among these 69 genes, *RTN4* exhibits the most significant association with TNBC in the TWAS, and its expression is significantly downregulated in TNBC across all comparisons. Our findings highlight a set of potential tumor suppressor genes that are downregulated in TNBC and may play crucial roles in its development and progression, offering promising targets for future therapeutic interventions.

### Identification of novel, differentially expressed tumor promoter genes in basal-like and claudin-low TNBC

We performed a transcriptomic analysis of TNBC and normal breast tissue using TCGA and GTEx data. Focusing on genes with a positive effect size in TNBC and a *p* < 0.05, we identified potential TNBC-promoting genes. These genes displayed distinct expression patterns when comparing claudin-low and basal-like TNBC subtypes to normal breast tissues from both GTEx and TCGA (Fig. 4A).

**Fig. 5:**
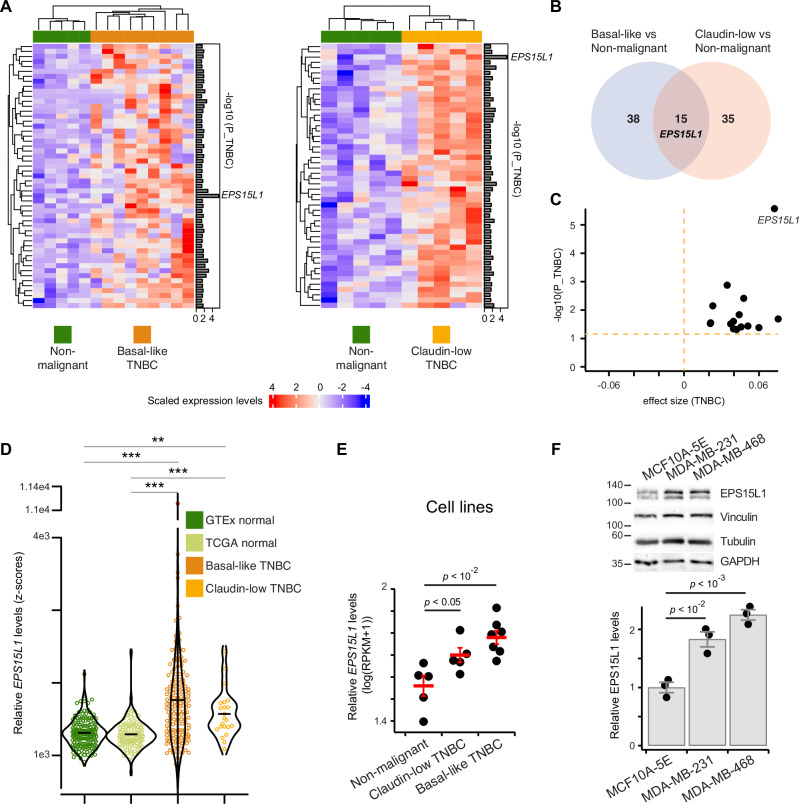
Suitability of MCF10A-5E (immortalized, nontumorigenic), MDA-MB-231 (claudin-low TNBC), and MDA-MB-468 (basal-like TNBC) cell lines for evaluating EPS15L1 function. **A** Heatmaps of pro-TNBC genes differentially expressed between immortalized, non-malignant breast epithelial cell lines (184A1, 184B5, MCF10A, MCF10F, MCF12A) and basal-like TNBC cell lines (HCC1143, HCC1599, HCC1806, HCC1937, HCC3153, HCC70, MB157, SUM149PT, SUM229PE) (left), or claudin-low TNBC cell lines (BT549, HCC1395, HCC38, HS578T, MDA-MB-231) (right). Genes were hierarchically clustered. The number of differentially expressed genes was *n* = 53 (basal-like vs non-malignant) and *n* = 50 (claudin-low vs non-malignant). **B** Venn diagram showing overlap of differentially expressed pro-TNBC genes between the two comparisons in (**A**); EPS15L1 is shared. **C** Volcano plot of the 15 pro-TNBC genes consistently upregulated across TNBC subtype cell-line panels. *x*-axis: TWAS effect size (TNBC). *y*-axis: −log10(P_TNBC). The horizontal dashed line marks P_TNBC = 0.05 and the vertical dashed line marks effect size (TNBC) = 0. EPS15L1 is highlighted. **D** EPS15L1 expression in patient tissue cohorts: GTEx normal breast (*n* = 179), TCGA solid normal breast (*n* = 113), TCGA basal-like TNBC (*n* = 162), and TCGA claudin-low TNBC (*n* = 22). Violin plots show distributions; dots represent individual samples; black bars denote the mean. Differential-expression significance is reported as Benjamini–Hochberg FDR–adjusted *p*-values. **E** EPS15L1 expression across epithelial and TNBC cell-line panels (as in **A**). RPKM values derived from aligned reads are shown as log(RPKM + 1). Dots indicate individual cell lines; red bars indicate mean ± SEM. *P*-values are annotated on the plot (Welch’s two-sided t-test; pairwise comparisons vs non-malignant). **F** Immunoblot of EPS15L1 in MCF10A-5E, MDA-MB-231, and MDA-MB-468 cells; vinculin, tubulin, and GAPDH are loading controls. Quantification is shown as mean ± SEM with Welch’s two-sided *t*-test *p*-values annotated.

Following manual curation, we identified 56 potential TNBC promoter genes, which were significantly upregulated in both TNBC subtypes compared to normal tissues (Fig. 4A–D and S5). Among these genes, functional annotations highlight roles in regulating EGFR endocytosis and signaling. Several genes are implicated in promoting EGFR stabilization, translocation, and endosomal trafficking. Additionally, many genes are involved in controlling EGFR signaling pathways, acting as co-activators or feedback regulators. These genes also enhance EGFR-driven progression and metastasis, making them potential therapeutic targets in breast cancer. These genes include *CBX3*^61^, *SEC61B*^62^, *AP1B1*^63^, *ARSB*^64^, *CHMP1A*^65^, *EDEM2*^66^, *RELB*^67^, *RHOD*^68^, *SERPINE2*^69^, *PTPRO*^70^, *RAB13*^71^, *ABL2*^72–75^, and *EPS15L1*^41,42^ (Table S2). Of particular note, *EPS15L1* showed the strongest association with TNBC, displaying the smallest *p*-value in the TWAS (Figs. 2A–D, 4A, 5D, and S6). *EPS15L1* drives residual EGFR-clathrin-mediated endocytosis in TNBC cells and mediates cholesterol-induced epithelial-to-mesenchymal transition through EGFR stabilization in prostate cancer cells^41,42^. These findings highlight *EPS15L1* as a key regulator of EGFR signaling and a potential therapeutic target in TNBC where EGFR is dysregulated.

### Single-cell characterization of EPS15L1 in TNBC tumors

To determine which cell types express *EPS15L1* in TNBC, we re-analyzed single-cell RNA-seq data from three TNBC tumors (E-MTAB-8107 dataset)^76^. After quality control and clustering, visualization plots (UMAP, t-SNE, and PCA) clearly separated the cells into three main groups for all patients: malignant-like epithelial, immune, and stromal cells (Fig. S7A–C). We identified each cell group by checking for known marker genes for epithelial (EPCAM, KRTs), immune (T cell, B cell, and myeloid markers), and stromal (VIM, collagens) cells. This process confirmed a distinct malignant cell group. We then checked EPS15L1 expression and found it was strongly and specifically expressed in the malignant-like epithelial cells, but not in the immune or stromal cells (Fig. S7D). This finding shows that EPS15L1 expression is highly specific to the malignant cells within the TNBC tumor microenvironment.

### TNBC cell lines as suitable models for evaluating EPS15L1 function

We conducted transcriptomic analyses on cell lines representing basal-like TNBC, claudin-low TNBC, and non-malignant breast epithelial tissue^77^, focusing on genes that demonstrated a positive effect size in TNBC with a *p*-value of less than 0.05 (Fig. 2A–D). RNA-seq data revealed two clusters of TNBC-related genes that displayed distinct expression patterns when comparing either basal-like or claudin-low TNBC subtypes to immortalized, non-tumorigenic breast epithelial cells (Fig. 5A). We further identified a group of 15 transcripts significantly upregulated in both TNBC subtypes compared to non-tumorigenic tissues (Fig. 5B). Among these, *EPS15L1* emerged as a major determinant of mRNA levels in both TNBC subtypes, also exhibiting the lowest *p*-value for TNBC in the TWAS (Fig. 5C and 5E). Quantitative immunoblotting confirmed a significant increase in EPS15L1 protein levels in MDA-MB-468 (a basal-like TNBC cell line) and MDA-MB-231 (a claudin-low TNBC cell line) compared to MCF10A-5E (a non-malignant breast epithelial cell clone) (Fig. 5F)^7^. Overall, this panel of cell lines recapitulates the EPS15L1 upregulation observed in TNBC tumors and provides a suitable model for further functional characterization of EPS15L1.

### Generalizability of EPS15L1 expression across TNBC subtypes

To assess the generalizability of our findings, we examined EPS15L1 expression across refined TNBC classifications using external datasets and a diverse cell line panel.

**Fig. 6:**
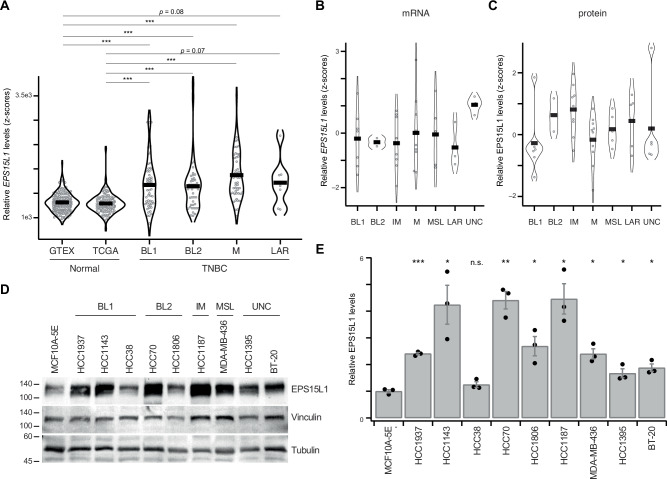
EPS15L1 is upregulated across diverse TNBC subtypes. **A** Analysis of EPS15L1 expression in TCGA TNBC subtypes stratified by Lehmann et al. (2021) compared to normal breast epithelial tissue from GTEx (*n* = 179) and TCGA (*n* = 113). TNBC subtypes included: basal-like 1 (BL1) (*n* = 60), basal-like 2 (BL2) (*n* = 45), mesenchymal (M) (*n* = 50), and luminal androgen receptor (LAR) (*n* = 7). **B**–**C** EPS15L1 mRNA and protein levels in the Anurag et al. (2022) cohort. Subtypes included BL1, BL2, immunomodulatory (IM), M, mesenchymal stem-like (MSL), LAR, and unclassified (UNC). **B** mRNA: BL1 (*n* = 7), BL2 (*n* = 2), IM (*n* = 8), M (*n* = 9), MSL (*n* = 5), LAR (*n* = 3), UNC (*n* = 4). **C** Protein: BL1 (*n* = 7), BL2 (*n* = 3), IM (*n* = 9), M (*n* = 10), MSL (*n* = 5), LAR (*n* = 5), UNC (*n* = 5). Representative immunoblot (**D**) and quantification (**E**) of EPS15L1 protein expression in MCF10A-5E controls versus a panel of TNBC cell lines. Cell lines represented BL1 (HCC1937, HCC1143, HCC38), BL2 (HCC70, HCC1806), IM (HCC1187), MSL (MDA-MB-436), and UNC (HCC1395, BT-20). Vinculin and tubulin served as loading controls. In (**A**–**C**), horizontal black bars indicate the mean expression. For (**A**) and (**E**), *p*-values were determined using Welch’s two-sided t-test.

First, we utilized data from Lehmann et al. (2021), who performed multi-omics characterization of TNBC subtypes—basal-like (BL1, BL2), mesenchymal (M), and luminal androgen receptor (LAR)—within the TCGA cohort^14^. Compared to normal breast epithelial tissue (GTEx and TCGA), EPS15L1 was significantly upregulated in BL1, BL2, and M subtypes (Fig. 6A). Although the LAR subtype had limited sample size (*n* = 7), EPS15L1 showed a trend toward upregulation (*P* = 0.08 and *P* = 0.07 versus GTEx and TCGA controls, respectively).

Second, we analyzed the proteogenomic dataset from Anurag et al. (2022), which classifies TNBC into BL1, BL2, immunomodulatory (IM), M, mesenchymal stem-like (MSL), LAR, and unclassified (UNC) subtypes^78^. EPS15L1 expression was comparable across all subtypes at both mRNA and protein levels (Fig. 6B–C).

Finally, we validated these findings by quantitative immunoblotting across TNBC cell lines representing diverse subtypes. EPS15L1 protein levels were significantly elevated compared to MCF10A-5E control in lines representing BL1 (MDA-MB-468, HCC1937, HCC1143), BL2 (HCC70, HCC1806), IM (HCC1187), MSL (MDA-MB-231, MDA-MB-436), and UNC (HCC1395, BT-20) subtypes (Figs. 5F and 6D–E). The BL1 line HCC38 exhibited EPS15L1 levels comparable to or exceeding the control. These data demonstrate that EPS15L1 upregulation is a consistent feature across the heterogeneous TNBC spectrum.

### EPS15L1 promotes growth in human TNBC cells

**Fig. 7:**
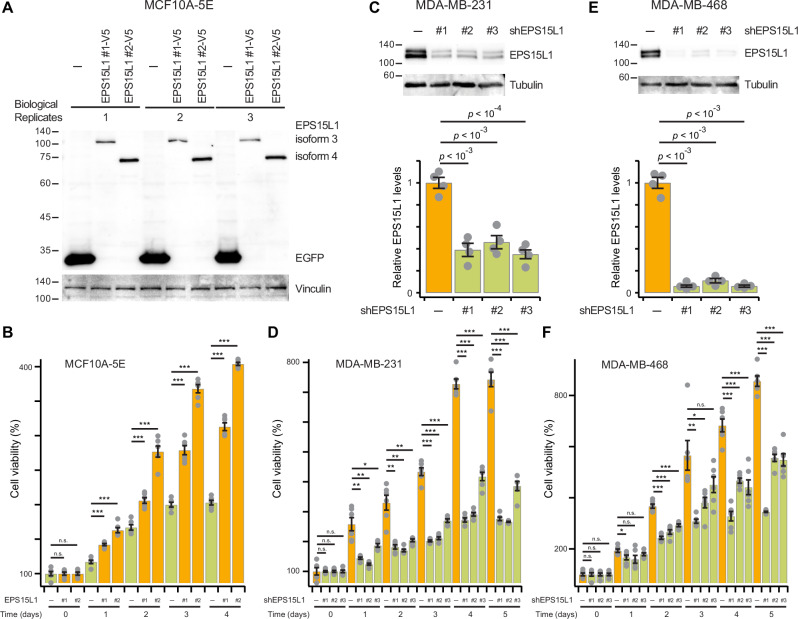
Evaluating EPS15L1 function in non-tumorigenic and TNBC cell lines. **A** Immunoblot confirmation of EPS15L1 overexpression in MCF10A-5E cells stably expressing EGFP-V5 control (–), EPS15L1-V5 #1 (transcript variant 3; isoform 3), or EPS15L1-V5 #2 (transcript variant 4; isoform 4). V5 was used to detect transgenes; vinculin served as a loading control. **B** Time-course cell viability of the cells in (**A**) measured by alamarBlue at the indicated time points. DOX-inducible shRNA knockdown of EPS15L1 in MDA-MB-231 (**C**) and MDA-MB-468 (**E**) cells using shLuc control (–) or shEPS15L1 #1–#3. EPS15L1 protein levels were assessed by immunoblotting; tubulin served as a loading control. Blots shown are representative of four independent biological replicates. EPS15L1 abundance was normalized such that the geometric mean in shLuc control cells equals 1. Exact P values are shown in C and E (Welch’s two-sided t-test). **D**, **F** Time-course cell viability of the cells in (**C**) and (**E**), respectively, measured by alamarBlue at the indicated time points. Data are shown as mean ± SEM from six independent biological samples. Statistical comparisons used Welch’s two-sided t-test; n.s., not significant; *P < 0.05, **P < 0.01, ***P < 0.001.

MCF10A-5E cells, clonally derived from non-tumorigenic epithelial MCF10A cells, exhibit homogeneous behavior in 3D cultures^79–85^. They form polarized acinar structures, undergo growth arrest, and do not exhibit aggressive phenotypes in 3D models. We used alamarBlue cell viability assays to assess cell proliferation by measuring metabolic activity as an indicator of viability. To investigate the impact of EPS15L1 on cell proliferation, we conducted time-course experiments from days 0 to 4 using alamarBlue assays. When engineered to express the coding sequence (CDS) of either EPS15L1 transcript variant 3 (EPS15L1 #1) or variant 4 (EPS15L1 #2) (obtained from DNASU), MCF10A-5E cells exhibited increased proliferation compared to control cells (Fig. 7A–B). This suggests that EPS15L1 expression confers a growth advantage to MCF10A-5E cells.

**Fig. 8:**
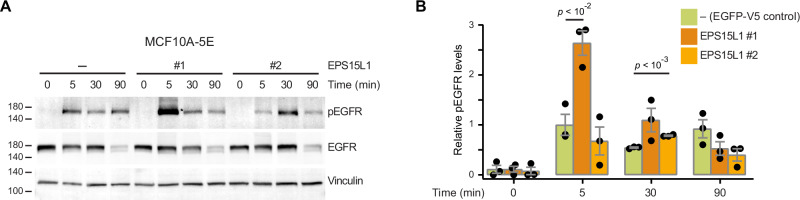
EPS15L1 isoforms differentially potentiate early EGFR phosphorylation without altering net EGFR downregulation. (**A** and **B**) MCF10A-5E cells stably expressing EGFP-V5 control (–), EPS15L1-V5 #1 (transcript variant 3), or EPS15L1-V5 #2 (transcript variant 4) were stimulated with EGF (100 ng/mL) for 0, 5, 30, or 90 min and analyzed by immunoblotting for total EGFR and phosphorylated EGFR. EGFR abundance was similarly reduced by 90 min across all conditions. In contrast, EPS15L1 enhanced early EGFR signaling with isoform-specific kinetics. Vinculin served as a loading control. P-values were calculated using Welch’s two-sided t-test.

EPS15L1 #1 and EPS15L1 #2 encode proteins (NP_001245304.1 and NP_001245305.1, respectively) that share an identical N-terminal region comprising three Eps15 homology (EH) domains and a coiled-coil core (aa 1–597) but diverge at the C-terminus: EPS15L1 #1 contains an extended, low-complexity, proline-rich tail harboring 16 DPF (Asp-Pro-Phe) motifs, whereas EPS15L1 #2 terminates shortly after the shared core and lacks the DPF-rich region entirely. Because DPF motifs mediate AP-2 α-adaptin engagement during clathrin-mediated endocytosis^86^, this structural difference predicts isoform-specific coupling to AP-2–dependent coat assembly. To examine signaling consequences, MCF10A-5E cells were stimulated with EGF (100 ng/mL) and analyzed at 0, 5, 30, and 90 min. EGFR abundance was similarly downregulated by 90 min in both control and EPS15L1-expressing cells, indicating no detectable effect on net receptor degradation (Fig. 8). However, both isoforms selectively potentiated early EGFR signaling with distinct kinetics: EPS15L1 #1 enhanced EGFR phosphorylation at 5 min (2.64-fold), whereas EPS15L1 #2 enhanced phosphorylation at 30 min (1.44-fold) relative to EGFP-V5 controls. These findings demonstrate isoform-specific modulation of EGFR signaling dynamics without alteration of overall receptor internalization, supporting a model in which both variants enhance proliferative signaling through amplification of early EGFR activation.

To assess whether EPS15L1 is essential for the survival of TNBC cells, we employed three doxycycline (DOX)-inducible shRNAs targeting non-overlapping sites within the CDS of EPS15L1 in MDA-MB-231 and MDA-MB-468 cells. Quantitative immunoblotting analysis confirmed successful knockdown of *EPS15L1* in DOX-treated cells (Fig. 7C and 7E). Growth inhibition associated with *EPS15L1* knockdown was consistently observed in time-course studies from days 0 to 5 (Fig. 7D and 7F). These results indicate that targeting *EPS15L1* expression could effectively inhibit growth in both basal-like and claudin-low TNBC subtypes. Our findings suggest that *EPS15L1* represents an actionable molecular vulnerability in TNBC cells.

To examine downstream signaling consequences, serum-starved MDA-MB-231 and MDA-MB-468 cells were stimulated with EGF (100 ng/mL) for 0–90 min. Total EGFR abundance remained largely stable in both lines (Fig. S8); the high receptor copy number in these cells saturates degradative machinery^87^. Although p-EGFR peaked at 5 min and declined thereafter, p-ERK1/2 was robustly induced at 5 min and sustained through 90 min (Fig. S8). EPS15L1 knockdown (shEPS15L1 #1 and #2) did not alter the kinetics or magnitude of EGFR or ERK1/2 phosphorylation. These results are consistent with the model that acute MAPK activation is predominantly driven by signaling at the plasma membrane and can be sustained by a small fraction of active receptors^88,89^. Because endocytic adapters often regulate spatiotemporal signal compartmentalization rather than initial phosphorylation amplitude, EPS15L1-dependent trafficking effects may be masked in whole-cell lysate measurements. These observations suggest that EPS15L1 influences long-term cellular phenotypes through mechanisms distinct from acute, plasma membrane-proximal signaling.

### EPS15L1 promotes growth of low-density TNBC cells in adherent 2D in vitro cultures

Unlike alamarBlue cell viability assays, which assess metabolic activity, reductive capacity, and cell energization, clonogenic assays specifically quantify the proliferative fraction of single cells capable of forming colonies comprising at least 50 cells. This serves as an indicator of long-term, ‘unlimited’ proliferation. The clonogenic potential is influenced by the unique growth requirements of cells isolated at low densities, which fundamentally differ from the requirements of those at higher densities.

**Fig. 9:**
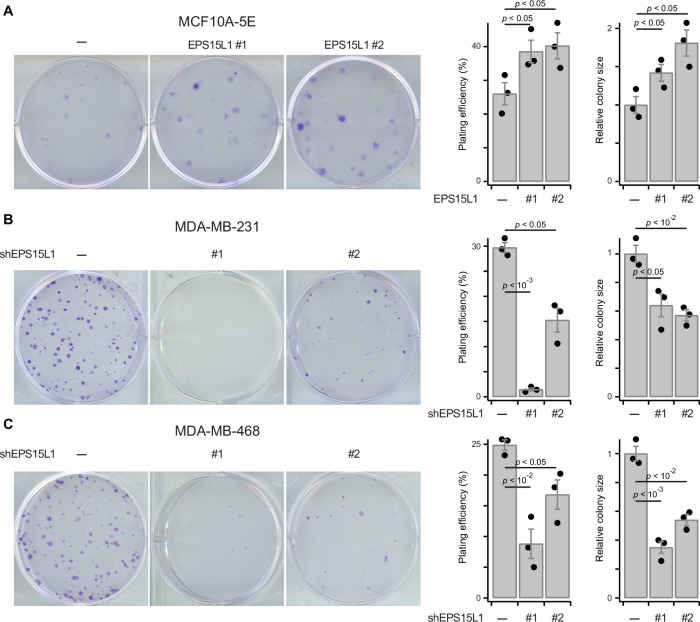
EPS15L1 promotes clonogenic outgrowth in mammary epithelial cells and supports clonogenicity of TNBC cells. **A** Representative crystal violet–stained clonogenic plates of MCF10A-5E cells stably expressing EGFP-V5 control (–), EPS15L1-V5 #1 (transcript variant 3), or EPS15L1-V5 #2 (transcript variant 4). Cells were seeded at 100 cells/well and cultured for 10 days. Right, quantification of plating efficiency (%) and relative colony size (normalized to the geometric mean of EGFP-V5 controls set to 1). Each dot represents an independent biological replicate; bars show mean ± SEM (*n* = 3). *P* values are shown; Welch’s two-sided t-test. Representative clonogenic plates of MDA-MB-231 (**B**) and MDA-MB-468 (**C**) cells expressing inducible shLuc control (–) or shEPS15L1 #1/#2. Cells were seeded at 500 cells/well and cultured for 13 days. Right, quantification of plating efficiency and relative colony size (normalized to the geometric mean of shLuc controls set to 1). Each dot represents an independent biological replicate; bars show mean ± SEM (*n* = 3). *P* values are shown; Welch’s two-sided *t*-test.

As demonstrated by Puck and Marcus, optimizing key parameters, including cell seeding density and colony-formation incubation time (allowing for at least six population doublings), is crucial for successful clonogenic assays^90^. We first optimized single-cell growth conditions for each cell type to achieve consistent plating efficiency (PE) and clonogenic growth with approximately 25–30% PE (Fig. 9). PE is defined as the number of colonies formed divided by the number of initially seeded single cells. For the assay, single-cell suspensions were generated from a donor culture and seeded into six-well plates, where they were allowed to adhere. We performed clonogenic assays on MCF10A-5E (100 cells/well for 10 days), MDA-MB-231, and MDA-MB-468 (500 cells/well for 13 days) cells, using three independent biological replicates for each cell line.

To investigate functional differences between control and EPS15L1-manipulated cells, we measured colony number and calculated PE. MCF10A-5E control cells exhibited a PE of 26.01%, while overexpression of EPS15L1 #1 and #2 resulted in PEs of 38.57% and 40.25%, respectively (Fig. 9A). MDA-MB-231 control cells had a PE of 29.73%, whereas EPS15L1 knockdown with DOX-inducible shEPS15L1 #1 and #2 reduced PE to 1.47% and 15.27%, respectively (Fig. 9B). Similarly, MDA-MB-468 control cells showed a PE of 24.87%, with shEPS15L1 #1 and #2 reducing PE to 8.8% and 16.80%, respectively (Fig. 9C).

We also measured the cell-covered area of stained colonies as an estimation of cell number per colony. Although colony sizes varied within each population, the average colony area was significantly larger in EPS15L1-high cells. MCF10A-5E cells overexpressing EPS15L1 #1 and #2 had relative colony sizes of 1.42- fold and 1.81-fold compared to control, respectively (Fig. 9A). Conversely, MDA-MB-231 cells with EPS15L1 knockdown (shEPS15L1 #1 and #2) had relative colony sizes of 64% and 57%, respectively, compared to control (Fig. 9B). Similarly, MDA-MB-468 cells with EPS15L1 knockdown (shEPS15L1 #1 and #2) showed relative colony sizes of 35% and 54% compared to control (Fig. 9C). Collectively, these results demonstrate that EPS15L1 enhances the ability of TNBC cells to form colonies when cultured as single cells in vitro on standard tissue culture plastic.

### Essential role of EPS15L1 in single-cell–initiated spheroid formation and protrusive outgrowth morphology of TNBC cells in a 3D microenvironment

Single-cell-derived spheroids can recapitulate tumor initiation, influenced by both interactions within the 3D microenvironment, which mimics physiological conditions, and the cells’ inherent genetic identity. MDA-MB-231 cell spheroids are a well-characterized in vitro TNBC model, effectively used for detecting malignant cells and tumorigenesis^91^. These spheroids maintain a compact and uniform structure for up to 9 days. Compared to monolayer cultures, they exhibit a slower growth rate. Previous studies have demonstrated that the number of Ki-67 positive cells, a proliferation marker, decreases after day 9, primarily localizing near the spheroid periphery^91^. We have previously observed that MDA-MB-231 spheroids can develop a stellate, protrusive outgrowth morphology during extended 3D culture^81,92^. This reproducible developmental pattern makes MDA-MB-231 spheroids a suitable model for studying TNBC growth and spheroid development under 3D conditions that incorporate extracellular matrix (ECM) context and isogenic neighboring cells.

**Fig. 10:**
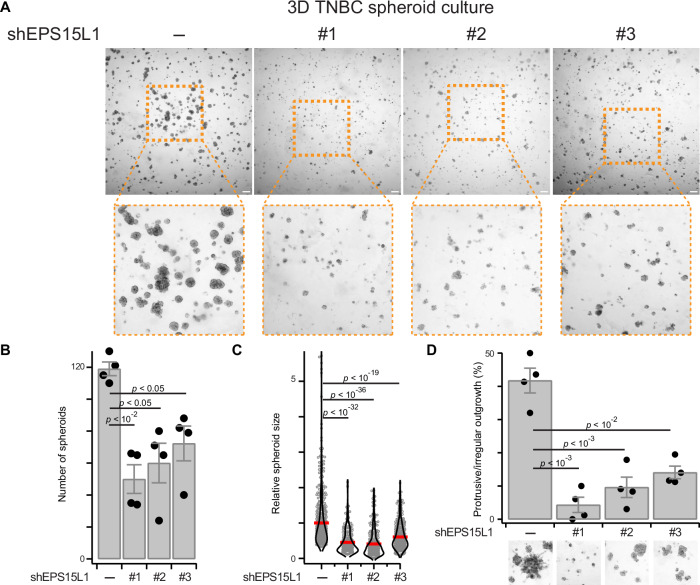
EPS15L1 promotes spheroid growth and protrusive outgrowth morphology of TNBC cells in 3D culture. **A**–**C** EPS15L1 knockdown inhibits spheroid formation and reduces spheroid size in MDA-MB-231 cells. MDA-MB-231 cells with stable EPS15L1 knockdown using three independent shRNAs (shEPS15L1 #1, #2, #3) were compared with control cells expressing a non-targeting shRNA (shLuc; “–”). **A** Representative bright-field images of 3D spheroids (boxed regions shown at higher magnification). **B** Quantification of spheroid formation (number of spheroids). **C** Distribution of relative spheroid size for shLuc (*n* = 476 spheroids), shEPS15L1 #1 (*n* = 200), shEPS15L1 #2 (*n* = 240), and shEPS15L1 #3 (*n* = 289); red horizontal line denotes the mean. Spheroid formation and size were assessed after 9 days of 3D culture. **D** EPS15L1 knockdown reduces the frequency of protrusive/irregular spheroid outgrowth in MDA-MB-231 3D cultures assessed at day 12. Representative examples are shown below the plot. Data in (**B**) and (**D**) are presented as mean ± SEM from four independent biological replicates. *P*-values were determined using Welch’s two-sided t-test.

We utilized MDA-MB-231 spheroids to explore the contribution of EPS15L1 to breast cancer progression. At day 9, control MDA-MB-231 spheroids maintained a compact structure. In contrast, EPS15L1 knockdown with shEPS15L1 #1, #2, and #3 resulted in significantly smaller and fewer spheroids, although with some heterogeneity (Fig. 10A–C). We extended the 3D culture to 12 days to investigate the effects of EPS15L1 knockdown on spheroid morphology. Compared to control cells, shEPS15L1 MDA-MB-231 cells (#1, #2, #3) exhibited a significantly reduced frequency of stellate/protrusive morphology, decreasing from 41.73% to 4.32%, 9.60%, and 14.08%, respectively (Fig. 10D). These data collectively suggest that EPS15L1 upregulation enhances MDA-MB-231 spheroid formation and promotes an aggressive morphological phenotype in single-cell-initiated TNBC spheroids.

### Association of EPS15L1 with poor prognosis in patients with TNBC

**Fig. 11:**
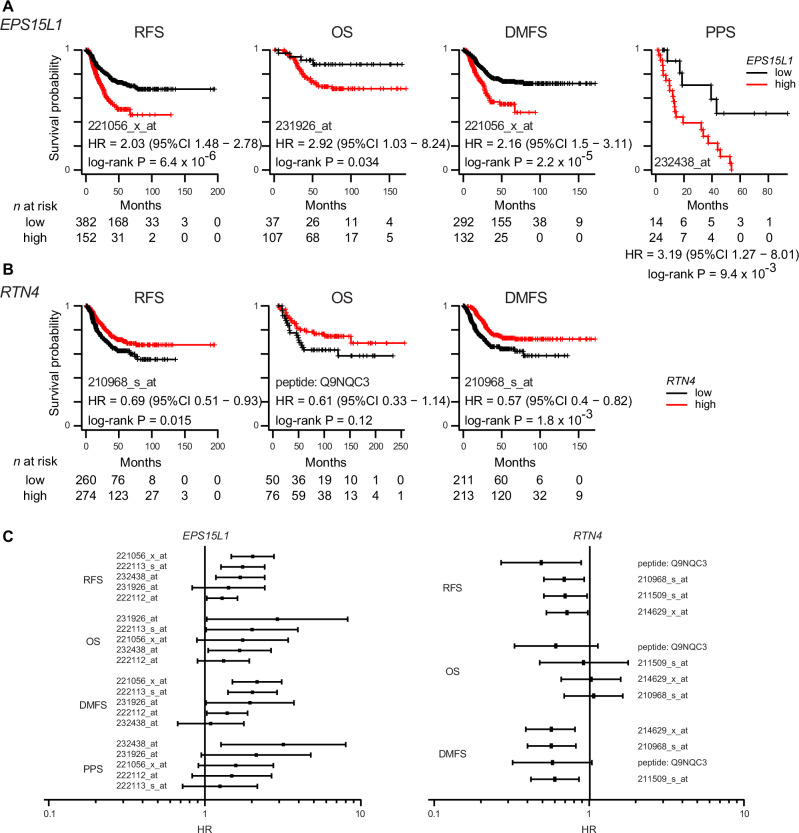
EPS15L1 and RTN4 expression is associated with survival outcomes in TNBC. Kaplan–Meier survival analyses of TNBC patients stratified by high (red) versus low (black) expression of EPS15L1 (**A**) or RTN4 (**B**) using the Kaplan–Meier plotter database. Endpoints include relapse-free survival (RFS), overall survival (OS), distant metastasis-free survival (DMFS), and post-progression survival (PPS; shown for EPS15L1 as indicated). Hazard ratios (HR) with 95% confidence intervals and log-rank *P* values are shown in each plot; tick marks indicate censored cases and numbers below plots indicate patients at risk. The expression probe/peptide identifiers are indicated within each plot; EPS15L1 analyses used Affymetrix probes (221056_x_at, 222112_at, 222113_s_at, 231926_at, 232438_at), and RTN4 analyses used Affymetrix probes (210968_s_at, 211509_s_at, 214629_x_at) and the RTN4 peptide (UniProt: Q9NQC3). **C** Summary forest plots of HRs derived from analyses using individual EPS15L1 probe IDs and RTN4 probe/peptide IDs across endpoints.

The prognostic role of the *EPS15L1* gene was examined using Kaplan-Meier survival analysis in independent cohorts of TNBC patients^93^. Elevated *EPS15L1* expression was associated with significantly reduced relapse-free survival (RFS) in 534 patients (Hazard Ratio (HR) = 2.03, *P* = 6.4 × 10⁻⁶), overall survival (OS) in 144 patients (HR = 2.92, *P* = 0.034), distant metastasis-free survival (DMFS) in 424 patients (HR = 2.16, *P* = 2.2 × 10⁻^5^), and post-progression survival (PPS) in 38 patients (HR = 3.19, *P* = 9.4 × 10⁻^3^) (Fig. 11A). These associations were confirmed using additional *EPS15L1* probes, indicating that high *EPS15L1* expression is associated with poor clinical outcomes in TNBC (Figs. 11C and S9).

In contrast, the *RTN4* gene, identified as a major TNBC suppressor (Fig. 3C–D), exhibited a contrasting prognostic impact. Elevated *RTN4* expression was associated with significantly improved RFS in the cohort of 534 patients (HR = 0.69, *P* = 0.015) (Fig. 11B). Additionally, OS and DMFS were notably better in patients with high RTN4 expression, with HRs of 0.61 (*P* = 0.12, *n* = 126) and 0.57 (*P* = 1.8 × 10⁻^3^, *n* = 424), respectively. Further analysis with additional *RTN4* probes supported the association between low *RTN4* expression and poor RFS and DMFS in TNBC patients (Figs. 11C and S10).

These results are consistent with our genome-wide analysis and suggest that *EPS15L1* upregulation contributes to the aggressive behavior of TNBC tumors, leading to a poor prognosis. Furthermore, they highlight the clinical significance of *EPS15L1* upregulation as a prognostic factor for poor survival in TNBC patients.

## Discussion

A central contribution of this study is a human-first prioritization strategy: we used genetically regulated expression to nominate candidate TNBC risk genes and then established functional relevance in TNBC models. This framework helps de-risk target nomination compared with expression-only approaches, but it does not by itself prove causality. TWAS signals can reflect linkage disequilibrium, co-regulation, tissue mismatch, or context-dependent eQTL architectures, and therefore require orthogonal validation and mechanistic follow-up to identify the true effector gene(s) at a locus^25,33^. In addition, our discovery phase relied on bulk breast tissue transcriptomes, which may be confounded by variable tumor purity and immune/stromal admixture. These factors can inflate apparent differential expression by shifting cell-type composition rather than changing malignant epithelial programs^94,95^. We addressed this limitation by validating EPS15L1 expression and function in epithelial-only cell line systems, supporting a tumor-cell–intrinsic requirement. Future work should further strengthen cellular attribution using deconvolution (e.g., ESTIMATE/CIBERSORT-like approaches^96^) and single-cell/spatial transcriptomics to define the malignant versus microenvironmental sources of EPS15L1 expression and to test whether EPS15L1 dependency associates with specific tumor states or microenvironmental contexts^94,95,97^.

EGFR pathway activation is frequently associated with aggressive behavior in TNBC, yet EGFR-directed antibodies and tyrosine kinase inhibitors (TKIs) have shown limited benefit in unselected TNBC cohorts, consistent with intrinsic and adaptive resistance mechanisms^35,98^. One implication is that EGFR abundance alone may be an imperfect biomarker for dependency: pathway output can be buffered by network redundancy, altered receptor trafficking, and context-specific rewiring during progression and metastasis^98^. Our data support an alternative therapeutic logic—targeting an upstream “signal-handling” module that shapes receptor availability and signaling geography rather than attempting to inhibit the receptor kinase directly. Endocytic trafficking is increasingly appreciated as a determinant of signaling duration, compartmentalization, and downregulation for many receptor systems; accordingly, dysregulation of trafficking can sustain oncogenic signaling without requiring activating receptor mutations^99,100^. By nominating and validating EPS15L1, we highlight endocytic control as a potentially actionable layer of TNBC biology that sits between receptor abundance and downstream pathway execution.

Mechanistically, EGFR internalization occurs through multiple routes with distinct signaling consequences. At low ligand doses, clathrin-mediated endocytosis (CME) commonly supports receptor recycling and signaling persistence, whereas higher ligand stimulation can engage non-clathrin endocytosis (NCE) and promote lysosomal sorting, thereby attenuating signaling^41,101^. EPS15-family proteins participate early in coated-pit assembly and cargo capture and can influence how efficiently receptors are internalized and sorted. Recent work further supports that clathrin-coated pits are not uniform; molecularly distinct pit populations can bias EGFR toward divergent fates and signaling outputs^102^. Within this conceptual framework, EPS15L1 overexpression in TNBC provides a plausible mechanism to amplify or prolong EGFR pathway activity by tuning the kinetics and routing of receptor trafficking rather than changing EGFR gene dosage. Importantly, endocytic adapters may predominantly affect spatial and temporal signaling (e.g., endosomal versus plasma membrane signaling pools) rather than the initial phosphorylation amplitude detectable in bulk lysates^99,100^. Thus, even when acute whole-lysate phospho-signals appear similar, altered trafficking can still produce meaningful differences in long-term transcriptional programs, proliferative fitness, and therapy response—phenotypes that are more directly captured by our growth, clonogenic, and 3D spheroid assays.

Placing EPS15L1 within the established TNBC driver landscape further underscores its distinct contribution. Core genomic drivers of TNBC—TP53 mutations ( > 80–90%), MYC amplification (∼40–50%), PTEN loss, RB1 deletion, and PIK3CA/AKT pathway alterations (10–30% across subtypes)—primarily confer genomic instability, defective DNA repair, cell-cycle dysregulation, and hyperactivation of PI3K/AKT-mTOR signaling^103^. These alterations are largely tumor-cell intrinsic and represent foundational oncogenic events that are necessary but often insufficient alone for full malignant transformation and therapeutic resistance. In contrast, EPS15L1 acts at a distinct regulatory node by governing EGFR endocytosis and trafficking, thereby controlling the intensity and duration of EGFR-RAS-RAF-MEK-ERK and, indirectly, PI3K/AKT signaling without requiring genetic alteration of EGFR itself. High EGFR protein expression or pathway activity is a hallmark of basal-like TNBC (BL1/BL2 subtypes) even in the absence of EGFR amplification or mutation^10^, indicating that post-translational amplification of EGFR signaling constitutes a critical parallel layer of oncogenesis. Thus, EPS15L1 overexpression represents an additive rather than redundant mechanism: it amplifies an already dysregulated EGFR axis that is not directly addressed by the canonical genomic drivers. This additive role is functionally evidenced by our data showing that EPS15L1 knockdown profoundly impairs growth, clonogenicity, and spheroid formation even in TNBC cells harboring TP53 mutation. Furthermore, synergistic potential is suggested by the convergence of EPS15L1-mediated EGFR trafficking with downstream PI3K/AKT hyperactivation driven by PIK3CA mutation or PTEN loss; combined inhibition of EGFR endocytosis and PI3K/AKT may therefore elicit synthetic lethality or overcome adaptive resistance. EPS15L1 consequently provides a previously unrecognized, therapeutically actionable layer atop the established genomic landscape of TNBC.

Several aspects of this work also delimit the current scope and motivate specific next steps. Functional validation was performed in vitro (2D viability, clonogenic outgrowth, and 3D single-cell–initiated spheroid formation). While these complementary assays capture key tumorigenic properties, the metastatic cascade is governed by systemic and tissue-specific determinants that require orthotopic and metastatic in vivo validation. In parallel, our prognostic analyses incorporate retrospective public datasets that are heterogeneous in treatment exposure, follow-up duration, and clinical annotation; accordingly, survival associations should be interpreted as supportive rather than definitive evidence of clinical utility. Notably, EPS15L1 was nominated through a human-first framework: TWAS prioritized EPS15L1 based on genetically regulated expression associated with TNBC risk, and independent analyses in TCGA and external survival cohorts linked elevated EPS15L1 expression to adverse clinical outcomes. This convergence of human genetic evidence, patient prognostic associations, and mechanistic in vitro phenotyping supports EPS15L1 as a clinically relevant TNBC vulnerability. Future studies should test EPS15L1 dependency in orthotopic mammary fat pad or intraductal xenograft models using inducible EPS15L1 suppression, and in metastatic paradigms (spontaneous and experimental dissemination assays) to quantify effects on colonization and therapeutic response.

From a translational perspective, EPS15L1 is not a classical drug target: as a non-enzymatic endocytic adapter lacking a catalytic active site, it functions primarily through protein–protein interactions, and no EPS15L1-selective pharmacological inhibitor currently exists. Nonetheless, multiple advanced therapeutic modalities could be leveraged to suppress EPS15L1 function. RNA-based approaches, such as small interfering RNA (siRNA) formulated in lipid nanoparticles (LNPs)^104^, provide a clinically validated route to gene silencing, contingent on achieving tumor-selective delivery. Targeted protein degradation strategies may be especially well-suited for scaffolding proteins: Proteolysis-Targeting Chimeras (PROTACs) or engineered “bioPROTAC” degraders can eliminate the functional complex by inducing ubiquitin-dependent proteasomal degradation^105^. Clathrin-mediated Endocytosis Targeting Chimeras (CleTACs) represent another rational approach^106^, leveraging endocytic machinery to drive lysosomal degradation of the target. The modular architecture of EPS15L1—particularly its EH domains that recognize NPF motifs—also creates an opportunity for peptidomimetic disruption; notably, high-affinity cyclic peptide inhibitors have been developed for related EH-domain interactions^107^, establishing a structural precedent for targeting these interfaces. Finally, unbiased chemogenomic screening could identify chemical starting points; for instance, independent multi-omics analyses in melanoma predicted an in silico interaction between EPS15L1 and doxorubicin^108^, providing a hypothesis-generating foothold for structure-guided ligand discovery in TNBC.

Important barriers to clinical translation must also be recognized. EPS15L1 operates within core endocytic machinery and exhibits partial functional redundancy with its paralog EPS15^109^, particularly in housekeeping pathways such as transferrin receptor endocytosis, which may narrow the therapeutic window. Moreover, genetic evidence from knockout studies indicates essential, non-redundant roles for EPS15L1 in synaptic vesicle recycling and neurodevelopment, raising concerns regarding on-target neurotoxicity^109^. These considerations argue that EPS15L1 is best advanced using selective, delivery-enabled precision modalities, with rigorous evaluation of redundancy, tissue liabilities, and on-target safety.

Collectively, our results support a model in which EPS15L1 represents a TNBC vulnerability at the level of receptor trafficking control. Beyond the specific gene, this work reinforces a broader concept: cancers may sustain oncogenic signaling not only through mutations in receptors and kinases, but also through dysregulation of the cellular logistics that govern receptor routing, residence time, and signaling compartmentalization. Systematically integrating human genetics with functional perturbation and trafficking-resolved mechanistic assays may therefore reveal additional, therapeutically addressable “signal-handling” nodes in TNBC and other receptor-driven malignancies.

## Methods

### Construction of gene expression prediction models

Our gene expression prediction model for breast mammary tissue was developed using eQTL summary statistics derived from the GTEx project^110^, as made available by the eQTL Catalog^44^. The eQTL Catalog provides a comprehensive workflow encompassing RNA-seq quantification, gene expression quality control (QC) and normalization, genotype QC and imputation, plus association testing and fine mapping. We focused on eQTLs with minor allele frequencies (MAF) of at least 1% and an imputation score of 0.3 or higher, based on the imputation quality metrics from the UK Biobank. For each gene, we employed a step-wise model selection process using GCTA to construct a joint model for gene expression prediction^111^. eQTLs demonstrating P-values of 0.05 or less in this joint model were retained. Their effect sizes were then incorporated as weights in the prediction model for both PrediXcan and S-PrediXcan methods. Specifically for S-PrediXcan, we calculated the covariance of the eQTLs using European sample data from the 1000 Genomes Project^112^.

### Study samples

We used the GWAS summary statistics for TNBC provided by the Breast Cancer Association Consortium (BCAC). These statistics were calculated based on a meta-analysis that combined the BCAC TNBC and the CIMBA results. The analysis included a total of 16,592 TNBC cases and 103,901 controls^16^. We also used the GWAS summary statistics for overall breast cancer provided by BCAC, calculated based on 133,384 breast cancer cases and 113,789 controls. From the UK Biobank, we identified 13,631 overall breast cancer cases and 138,289 controls in females. The overall breast cancer cases in the UK Biobank were identified using ICD-9 and ICD-10 codes from the cancer registry or self-reported cancer codes. The ICD-10 codes for overall breast cancer included those starting with C50 and its subclasses, while the ICD-9 codes included those beginning with 174 and its subclasses. Additionally, the self-reported cancer code 1002 was used. The GWAS data from the UK Biobank consisting of 10,264,766 genotyped and imputed SNPs with MAF ≥ 1% and imputation score ≥ 0.3 were used.

### Transcriptome-wide association analysis (TWAS)

Our TWAS employed the PrediXcan^24^ and S-PrediXcan^28^ approaches. PrediXcan is a computational tool designed to predict gene expression levels from an individual’s genotype data^113^. It utilizes pre-trained models based on genetic and transcriptomic data to infer gene expression in various tissues. This method allows for the investigation of the relationship between predicted gene expression and disease traits, providing insights into potential biological mechanisms. S-PrediXcan, on the other hand, extends the capabilities of PrediXcan by enabling transcriptome-wide association studies using only summary-level GWAS data. This approach does not require individual-level genotype data, making it more broadly applicable, especially in cases where only GWAS summary statistics are available. S-PrediXcan integrates the strengths of PrediXcan’s gene expression prediction models with GWAS summary statistics, offering a powerful tool for identifying gene-trait associations. In our study, S-PrediXcan was used to analyze summary statistics from the BCAC for both TNBC and overall breast cancer. Meanwhile, PrediXcan analyses were conducted using individual-level genotype data from the UK Biobank, focusing specifically on overall breast cancer. A meta-analysis was then performed to combine the two TWAS results for overall breast cancer, using the inverse-variance method based on a fixed-effects model^111^. Summary figures, including Manhattan plots, Q-Q plots, and lambda values (genomic inflation factors), were generated for each trait using the qqman R package (v0.1.9).

### Cell lines

The MCF10A-5E clone, derived from the parental MCF10A cell line, has been previously reported^79–81,83,85^. These cells are cultured in Dulbecco’s Modified Eagle’s Medium/Nutrient Mixture F-12 (Gibco) supplemented with 5% horse serum (Gibco), 20 ng/mL animal-free recombinant human epidermal growth factor (EGF) (Peprotech), 10 μg/mL insulin from bovine pancreas (Sigma), 0.5 μg/mL hydrocortisone (Sigma), 100 ng/mL cholera toxin from *Vibrio cholera* (Sigma), and 1x penicillin and streptomycin (Gibco). This female cell line is maintained at 37 °C in a 5% CO_2_ humidified incubator. MDA-MB-231 and MDA-MB-468 cells, both obtained from the American Type Culture Collection (ATCC), are cultured in Leibovitz’s L-15 medium (Gibco) with 10% fetal bovine serum (Gibco) and 1x penicillin and streptomycin (Gibco). These female cell lines are grown at 37 °C in a 100% air humidified incubator. The following cell lines were obtained from ATCC and cultured according to ATCC guidelines. All media were supplemented with 10% fetal bovine serum (FBS) and 1× penicillin-streptomycin (Gibco). HCC1937, HCC1143, HCC38, HCC70, HCC1806, HCC1187, and HCC1395 cells were maintained in RPMI-1640 medium. BT-20 cells were cultured in Eagle’s Minimum Essential Medium (EMEM). These cell lines were incubated at 37 °C in a humidified atmosphere containing 5% CO₂. MDA-MB-436 cells were maintained in Leibovitz’s L-15 medium supplemented with 10 µg/ml insulin and 16 µg/ml glutathione, and incubated at 37 °C in a humidified 100% air atmosphere. 293 T/17 cells, obtained from ATCC, are maintained in DMEM high glucose medium (Gibco) supplemented with 10% fetal bovine serum (Gibco) and 1x penicillin and streptomycin (Gibco). This cell line is cultured at 37 °C in a 5% CO_2_ humidified incubator. All cell lines are routinely tested and confirmed to be free of mycoplasma contamination.

### Plasmids

Tet-pLKO-puro (Addgene #21915)^114^, pDONR221_EGFP (Addgene #25899), pLX304 (Addgene #25890), pLX304-EPS15L1 #1 (DNASU, Clone ID: HsCD00437043, Insert sequence: 2263 nts, CDS of NM_001258375.2, EPS15L1 #1), pENTR223-EPS15L1-V5 #2 blast (DNASU, Clone ID: HsCD00516151, Insert sequence: 1804 nts, CDS of NM_001258376.2, EPS15L1 #2)^115^ were obtained commercially.

The shRNA target sequences for *EPS15L1* and luciferase, sourced from the RNAi Consortium shRNA Library, were as follows: shLuc: CTTCGAAATGTCCGTTCGGTT (TRCN0000072243); shEPS15L1 #1: GAGCATGCCACCGCCTAAATT (TRCN0000233084); shEPS15L1 #2: AGTCTGGCCTCTCGGACATTA (TRCN0000233083); shEPS15L1 #3: GAAGTCAACGCAAGACGAAAT (TRCN0000233085). shLuc and shEPS15L1 sequences were cloned into the Tet-pLKO-puro vector. The XhoI restriction site in the shRNA loop (CTCGAG) was altered to a PstI site (CTGCAG). Oligonucleotides were initially heated at 95 °C for 5 min in annealing buffer (10 mM Tris-HCl, pH 7.5, 100 mM NaCl, and 1 mM EDTA), then allowed to cool slowly to room temperature for annealing. The annealed primers were phosphorylated using T4 polynucleotide kinase (NEB #M0201) and subsequently cloned into the Tet-pLKO-puro vector previously digested with EcoRI and AgeI. For EPS15L1 expression experiments, pDONR221_EGFP (control) and pENTR223-EPS15L1 #1 were recombined with pLX304 using Gateway LR recombination (Invitrogen) to generate pLX304-EGFP-V5 blast and pLX304-EPS15L1-V5 #1 blast, respectively. All plasmids were confirmed by sequencing.

### Lentiviral production and cell transduction

Lentiviruses were produced in 293 T/17 cells by triple transfection of the lentiviral vector (Tet-pLKO-puro or pLX304), the packaging plasmid psPAX2 (Addgene #12260), and the envelope plasmid pMD2.G (Addgene #12259), as previously described^79,83,116–121^. Briefly, 1.25 μg of the lentiviral vector, 0.75 μg of psPAX2, and 0.5 μg of pMD2.G were mixed with 10 μL of 2.5 M CaCl_2_. 0.1 × TE (pH 7.6) was added to the mixture to make a final volume of 100 μL. This mixture was then added to 100 μL of 2 × × HEPES-buffered saline and incubated for 1 min. The resulting solution was added dropwise to a well of 293 T/17 cells. After 4–6 h of incubation at 37 °C, the medium was replaced with 1 mL of fresh growth medium. Viral supernatant was collected at 24 and 48 h post-transfection, sterile-filtered, and stored. Target cells were transduced with 500 μL of lentiviral supernatant in culture medium containing 8 μg/mL polybrene. Transduced cells were selected with puromycin (2 μg/mL) or blasticidin (10 μg/mL) until non-transduced control cells were eliminated.

### EGF stimulation time course

For EGF stimulation experiments, cells (MCF10A-5E cells stably expressing EGFP-V5, EPS15L1 #1, or EPS15L1 #2; and MDA-MB-231 and MDA-MB-468 cells expressing doxycycline-inducible shLuc or shEPS15L1 #1–#3) were seeded in 60-mm dishes and grown to 80–90% confluence. Cells were serum-starved overnight in basal medium lacking EGF and growth supplements. Cells were then stimulated with recombinant human EGF (100 ng/mL) for 0, 5, 30, or 90 min at 37°C. At each time point, cells were immediately placed on ice, washed twice with ice-cold phosphate-buffered saline (PBS), and lysed in RIPA buffer supplemented with protease inhibitors (10 µg/mL aprotinin, 10 µg/mL leupeptin, 1 µg/mL pepstatin, and 1 mM PMSF) and the phosphatase inhibitor activated sodium orthovanadate (Na_3_VO_4_; 0.2 mM). Lysates were clarified by centrifugation at 21,000 × *g* for 15 min at 4 °C, and protein concentrations were determined using a BCA assay.

### Quantitative immunoblotting

Quantitative immunoblotting was performed as previously described^79,83^. Briefly, protein samples were prepared in Laemmli sample buffer containing dithiothreitol (DTT) to a final volume of 20–40 μL. Samples were then separated by SDS-PAGE on 8%, 10%, or 12% polyacrylamide gels using Tris-glycine running buffer (25 mM Tris base, 250 mM glycine, 0.1% SDS) at 130 V. Proteins were transferred to PVDF membranes (Immobilon-FL, 0.45 μm pore size, Millipore) using a Mini Trans-Blot Electrophoretic Transfer Cell (Bio-Rad) in transfer buffer (24 mM Tris base, 190 mM glycine, 10% methanol) at 100 V for 1 h at 4 °C. Membranes were blocked with 0.5 × Intercept Blocking Buffer (LI-COR) for near-infrared fluorescent detection. Primary antibodies were diluted in 0.5 × Intercept T20 Antibody Diluent (LI-COR). PBS-based buffer was used for total protein detection, whereas TBS-based buffer was used for phosphorylated protein detection. The following primary antibodies were used: EPS15R (EP1147Y) (Abcam #ab53006, 1:50,000 dilution, RRID: AB_869661), Phospho-EGF Receptor (Tyr1068) (CST #2234, 1:1,000 dilution, RRID: AB_331701), EGF Receptor (CST #2232, 1:1,000 dilution, RRID: AB_331707), Phospho ERK1/2 (Thr202/Tyr204) (Clone D13.14.4E) (CST #4370, 1:2000 dilution, RRID: AB_2315112), ERK1/2 (CST #9102, 1:1,000 dilution, RRID: AB_330744), Vinculin (Clone V284) (Millipore #50-386, 1:10,000 dilution, RRID: AB_309711), GAPDH (Clone 6C5) (Ambion #AM4300, 1:10,000 dilution, RRID: AB_437392), GAPDH (6C5) (Merck # CB1001, 1:10,000 dilution, RRID: AB_2107426), Tubulin (Abcam #ab89984, 1:20,000 dilution, RRID: AB_10672056), and V5 Tag (Life Technologies #R960-25, 1:5,000 dilution, RRID: AB_2556564). After washing, membranes were incubated with secondary antibodies diluted in 0.5 × Intercept T20 (PBS) Antibody Diluent. Fluorescent signals were detected using a ChemiDoc MP Imaging System (Bio-Rad). Raw image exposures were analyzed using ImageJ software with the gel analysis tool.

### AlamarBlue cell viability assay

The alamarBlue cell viability assay was conducted using a homemade protocol as previously described^79^. Cells were seeded at the indicated densities in 90 μL of growth medium per well in 96-well plates. Each plate included three background control wells (90 μL of growth medium without cells) and three blank control wells (100 μL of growth medium without cells). Plates were incubated at 37 °C for the specified time. Then, 10 μL of 0.4 mg/mL resazurin solution (10 × ) was added to each well, except for the blank controls. Following a 2-h incubation at 37 °C, absorbance was measured at 570 nm and 595 nm using a microplate reader. The percentage reduction of alamarBlue was calculated as follows:

$${\rm{Percentage\; reduction\; of\; alamarBlue}}=({{\rm{A}}}_{{\rm{LW}}}-({{\rm{A}}}_{{\rm{HW}}}\times {{\rm{R}}}_{{\rm{o}}})\times 100)$$

where:

A_LW_ = absorbance at the lower wavelength

A_HW_ = absorbance at the higher wavelength

Correction factor R_o_ = AO_LW_/AO_HW_

AO_LW_ = absorbance of oxidized form of alamarBlue at the lower wavelength

AO_HW_ = absorbance of oxidized form of alamarBlue at the higher wavelength

### Clonogenic assay

To assess clonogenic potential, single-cell suspensions were prepared from a donor culture and seeded into six-well plates to allow cell adhesion. We optimized the single-cell growth conditions for each cell type to maintain consistent plating efficiency (PE) and clonogenic growth, achieving approximately 25–30% PE. Here, PE is defined as the ratio of the number of colonies formed to the number of initially seeded single cells. After seeding, the plates were incubated until the wells displayed sufficiently large colonies. Subsequently, the media was removed, and the cells were washed with 2 mL of PBS per well. Each well was then treated with 1 mL of fixing/staining solution composed of 4% paraformaldehyde (PFA) and 0.5% crystal violet in PBS for at least 30 min. Optionally, the fixing/staining solution could be filtered before application to prevent colony artifacts caused by crystal particles. After the staining period, the solution was carefully removed, and the dishes were rinsed with 2 mL of double-distilled water. The plates were then left to air dry at room temperature. Post-staining, colonies were counted, and the plates were photographed in their entirety. PE and colony size were determined for each treatment condition. The surviving fraction was calculated relative to control cells to assess treatment efficacy.

### 3D TNBC spheroid culture

Three-dimensional spheroid cultures were established using Matrigel (BD Biosciences) as previously described for MCF10A-5E cells^83^, with optimized culture conditions for the MDA-MB-231 cell line. Briefly, 45 μL of Matrigel was evenly distributed on the bottom of an 8-well chamber slide. For EPS15L1 knockdown experiments, stable cell lines were treated with 1 μg/mL doxycycline for 3 days prior to spheroid seeding. Single-cell suspensions were then seeded onto the Matrigel layer in Leibovitz’s L-15 medium supplemented with 10% fetal bovine serum, 1% penicillin-streptomycin, and 1 μg/mL doxycycline. Doxycycline was maintained in the 3D culture medium throughout the experiment. Culture medium was replaced every four days.

### RNA sequencing bioinformatics analysis

Gene expression data (mRNA expression z-scores) for breast tissue were downloaded from the UCSC Xena platform for TCGA and GTEx datasets^122^. These datasets included 179 normal breast tissue samples from GTEx, 113 solid normal breast tissue samples from TCGA, and 1092 primary breast tumor samples from TCGA. Primary breast tumor samples from TCGA were classified into the following subtypes: 499 Luminal A, 197 Luminal B, 171 normal-like, 78 HER2-enriched, and 193 TNBC, with some samples remaining unclassified, as previously described^8^. TNBC was further divided into 162 basal-like, 22 claudin-low, and 9 indistinguishable between basal-like and claudin-low subtypes. We analyzed mRNA expression z-scores for genes with a P-value < 0.05 for TNBC from our TWAS analysis. Genes were classified as “pro-TNBC” or “anti-TNBC” based on their effect size in the TNBC TWAS. Welch’s t-test was used to determine the statistical significance of differential gene expression between TNBC subgroups and normal breast tissue.

RNA sequencing data from the JWGray Breast Cancer Cell Line Panel were obtained from Synapse (ID: syn2346643), including whole-transcriptome shotgun sequencing of 53 breast cancer cell lines (JWGray_BCCL_counts_matrix_v1)^77^. The dataset comprises five immortalized, non-malignant breast epithelial cell lines (184A1, 184B5, MCF10A, MCF10F, MCF12A—previously classified as “normal-like” in Daemen et al.^77^), nine basal-like TNBC cell lines (HCC1143, HCC1599, HCC1806, HCC1937, HCC3153, HCC70, MB157, SUM149PT, SUM229PE), five claudin-low TNBC cell lines (BT549, HCC1395, HCC38, HS578T, MDA-MB-231), 22 ERBB2-amplified cell lines (21MT1, 21MT2, 21NT, 21PT, AU565, BT474, EFM192A, EFM192B, EFM192C, HCC1419, HCC1569, HCC1954, HCC202, HCC2218, JIMT1, MDA-MB-361, SKBR3, SUM190PT, SUM225CWN, UACC812, UACC893, ZR7530), and 12 luminal cell lines (BT483, CAMA1, HCC1428, LY2, MCF7, MDAMB175VII, MDA-MB-453, SUM52PE, T47D, ZR751, ZR75B, 600MPE). We analyzed mRNA expression data for genes significantly associated with TNBC (*p* < 0.05), focusing on pro-TNBC genes identified by positive effect sizes in the TNBC TWAS.

Relative gene expression was visualized using Igor Pro (WaveMetrics). Data analysis and visualization were primarily performed in the R programming environment (v.4.4.1) using the tidyverse package suite (v.2.0.0) and specialized visualization packages, including ComplexHeatmap (v.2.20.0)^123^.

### Single-cell RNA-seq processing and EPS15L1 expression analysis

#### Data source and patient selection

Single-cell RNA-seq data were obtained from the ArrayExpress database at EMBL-EBI (www.ebi.ac.uk/arrayexpress) under accession number E-MTAB-8107^76^. Three TNBC tumors (sc5rJUQ026, sc5rJUQ042, sc5rJUQ058) were selected for analysis. Gene–barcode count matrices were imported into R and processed using the Seurat package. Each cell was annotated with its corresponding patient ID and clinical information.

#### Quality control and filtering

Cells were assigned to specific patients based on sample barcode patterns. For each cell, we calculated the number of detected genes (*nFeature_RNA*), total UMI counts (*nCount_RNA*), and the percentage of reads mapping to mitochondrial (*percent.mt*) and ribosomal (*percent.ribo*) genes. Low-quality or stressed cells were excluded by keeping only those with more than 200 and fewer than 6000 detected genes, more than 500 total UMI counts, and less than 25% mitochondrial reads.

#### Normalization, feature selection, and dimensionality reduction

Filtered data were normalized using Seurat’s LogNormalize method with a scale factor of 10,000. Highly variable genes were identified using the variance-stabilizing transformation (VST) method, selecting the top 3000 features. All genes were scaled to have a mean of zero and unit variance. Principal component analysis (PCA) was performed using the variable genes, and the main principal components were used to build a shared nearest neighbor (SNN) graph. Nonlinear dimensionality reduction was performed using UMAP and t-SNE (based on the first 30 principal components) to visualize the transcriptional landscape across cells and patients.

#### Clustering and cell type annotation

Unsupervised clustering was conducted using Seurat’s FindNeighbors and FindClusters functions with an SNN graph and a resolution of 0.5. Cell types were annotated using both marker-based and module score–based approaches. Module scores were calculated from curated gene lists representing:

- Immune cells: CD3D, CD3E, CD8A, CD4, CD68, CD14, CD19, CD79A, NCAM1
- Malignant/epithelial cells: EPCAM, KRT8, KRT18, KRT19, KRT7, ESR1, PGR, ERBB2
- Stromal/endothelial cells: COL1A1, COL1A2, VIM, PDGFRA, ACTA2, PECAM1, VWF

Each cell was classified as Malignant, Immune, or Stromal according to its highest module score. Cell-type proportions were then summarized for each patient and cluster.

#### EPS15L1 expression quantification and visualization

Normalized expression values of *EPS15L1* were extracted and added to the Seurat metadata. Mean, median, and distribution statistics of *EPS15L1* expression were calculated for each annotated cell type, cluster, and patient. Average expression per (patient × cell type) group was visualized as heatmaps using ggplot2, allowing direct comparison of *EPS15L1* expression across malignant, immune, and stromal compartments.

### Patient survival analysis

Kaplan–Meier survival analysis was conducted in line with previously established methods^93^. We assessed the prognostic significance of *EPS15L1* and *RTN4* for patients with TNBC using the Kaplan–Meier Plotter database (http://kmplot.com/analysis/). This database includes survival data for breast cancer patients from the Gene Expression Omnibus, European Genome-Phenome Archive, and TCGA. For each gene symbol, Affymetrix probe IDs were inputted to generate KM plots. Patients were stratified into high and low expression groups based on the “auto select best cutoff” feature. We extracted data on relapse-free survival (RFS), overall survival (OS), distant metastasis-free survival (DMFS), and post-progression survival (PPS). Additionally, the number of cases, hazard ratios (HRs), and log-rank P-values were collated from the KM plotter to quantify the association between gene expression levels and patient outcomes.

### Ethics statement for publicly available human data

This study used publicly available, de-identified human datasets and did not involve direct interaction with human participants or the collection of identifiable private information. All analyses were performed in accordance with the Declaration of Helsinki and relevant institutional policies.

### Quantification and statistical analysis

For all experiments conducted in this study, details such as the sample size (n), the central tendency measure, and the dispersion metric are provided within the figure legends. The Kaplan-Meier survival curves were analyzed using the log-rank test. Two-sample comparisons were primarily carried out using Welch’s two-sided t-test. To ensure statistical robustness and account for multiple comparisons, we applied the Benjamini-Hochberg False Discovery Rate (FDR) correction for the gene expression analyses presented in Figs. 3D, 4D, and 5D. We did not employ any procedures to test the assumptions underlying parametric methods. All hypothesis tests were conducted with a type I error rate (α) set at 0.05, or an FDR-adjusted p-value < 0.05 where applicable.

### Use of a large language model for language editing

A large language model (ChatGPT, OpenAI) was used to assist with English-language editing and clarity (e.g., grammar, wording, and readability). All scientific content, interpretations, and final wording were reviewed and approved by the authors, who take full responsibility for the manuscript.

## Supplementary information

Supplementary Information

Supplementary Data 1

Supplementary Data 2

Supplementary Data 3

## Data Availability

The data analyzed in this study were obtained from public and controlled-access resources. Publicly available datasets used in this work include GTEx breast mammary tissue eQTL summary statistics via the eQTL Catalog (for gene expression prediction model construction); 1000 Genomes Project European reference genotypes (for linkage disequilibrium covariance estimation); and TCGA/GTEx breast tissue gene expression data accessed through the UCSC Xena platform (TCGA TARGET GTEx study; breast samples selected by primary site/sample type; gene expression dataset downloaded from UCSC Xena, https://xena.ucsc.edu/). We used the JWGray Breast Cancer Cell Line Panel RNA-seq dataset from Synapse (syn2346643), including whole-transcriptome shotgun sequencing data from 53 breast cancer cell lines (JWGray_BCCL_counts_matrix_v1), and single-cell RNA-seq data from ArrayExpress at EMBL-EBI under accession number E-MTAB-8107 (www.ebi.ac.uk/arrayexpress). Clinical data for patient survival analysis were obtained from the Kaplan–Meier Plotter database (http://kmplot.com/analysis/), which integrates datasets including E-MTAB-365, E-TABM-43, and GEO series GSE11121, GSE12093, GSE12276, GSE1456, GSE16391, GSE16446, GSE16716, GSE17705, GSE17907, GSE18728, GSE19615, GSE20194, GSE20271, GSE2034, GSE20685, GSE20711, GSE21653, GSE2603, GSE26971, GSE2990, GSE31448, GSE31519, GSE32646, GSE3494, GSE37946, GSE41998, GSE42568, GSE45255, GSE4611, GSE5327, GSE6532, GSE7390, and GSE9195. We also used external published datasets for cross-ancestry validation and TNBC subtype generalizability analyses, including African-ancestry TNBC GWAS summary statistics reported by Jia et al. (2024), and TNBC subtype datasets reported by Lehmann et al. (2021) and Anurag et al. (2022), as cited in the main text. BCAC GWAS summary statistics (including TNBC and overall breast cancer analyses, with the TNBC meta-analysis incorporating CIMBA) and UK Biobank data were used in accordance with the access conditions of those resources; UK Biobank individual-level data are available through application to UK Biobank and are subject to controlled-access and data-use restrictions. No new high-throughput sequencing datasets were generated in this study.
