# Supplementary material for: Identification and functional validation of EPS15L1 as a key driver of triple-negative breast cancer
Source: NPJ Breast Cancer. 2026 May 15;12:101. doi: 10.1038/s41523-026-00954-9 (PMC13434348; doi:10.1038/s41523-026-00954-9)
Supplement: Supplementary file 1 [file 41523_2026_954_MOESM1_ESM.pdf]

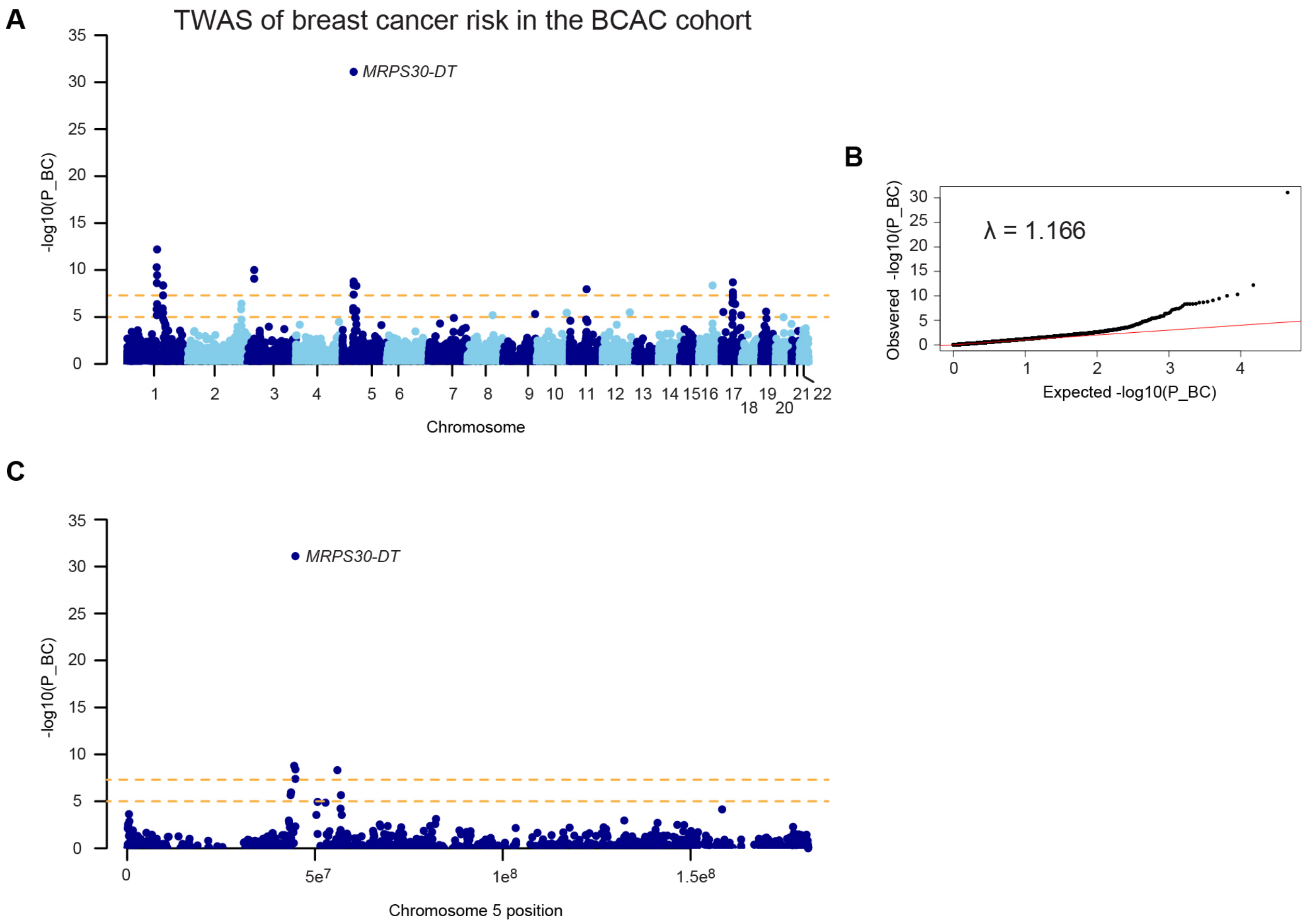


Figure S1. Transcriptome-wide association study (TWAS) of overall breast cancer risk using BCAC GWAS summary statistics.

(A) Manhattan plot of gene-level TWAS associations generated with S-PrediXcan using BCAC GWAS summary statistics. The x-axis indicates autosomal chromosomes (1–22), and the y-axis shows −log10(P_BC). Each point represents a tested gene (including non-coding transcripts where applicable); the top association (MRPS30-DT) is annotated. Dashed orange lines denote the significance thresholds ($P = 1 \times 10^{-5}$ and $P = 1 \times 10^{-8}$).

(B) Quantile–quantile (Q–Q) plot of observed versus expected −log10(P_BC) values for the TWAS results. The genomic inflation factor (λ) is indicated, and the red diagonal line represents the null expectation.

(C) Regional TWAS association plot across chromosome 5, showing −log10(P_BC) values by genomic position; dashed orange lines indicate the same thresholds as in (A).

(BCAC, Breast Cancer Association Consortium; GWAS, genome-wide association study.)

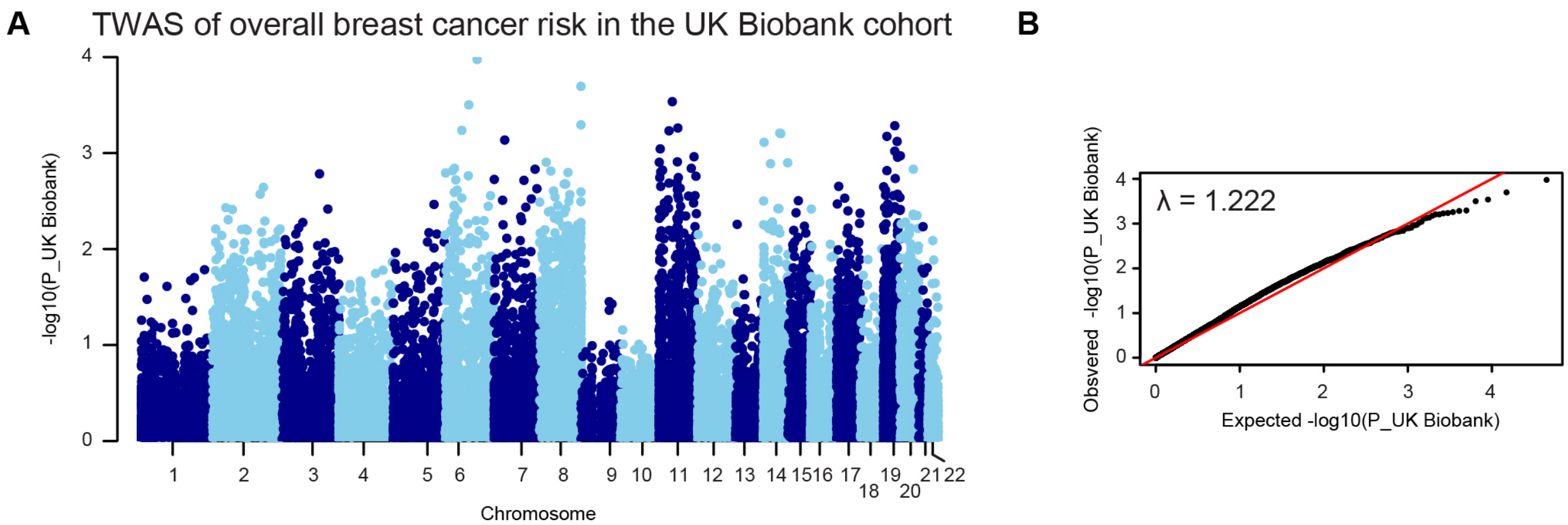


Figure S2. Transcriptome-wide association study (TWAS) of overall breast cancer risk in the UK Biobank cohort.

(A) Manhattan plot of gene-level TWAS association results in the UK Biobank cohort. The x-axis shows autosomal chromosomes (1–22), and the y-axis shows −log10(P_UK Biobank). Each point represents a tested gene.

(B) Quantile–quantile (Q–Q) plot of observed versus expected −log10(P_UK Biobank) values for the TWAS results. The genomic inflation factor (λ) is shown, and the diagonal line indicates the null expectation.

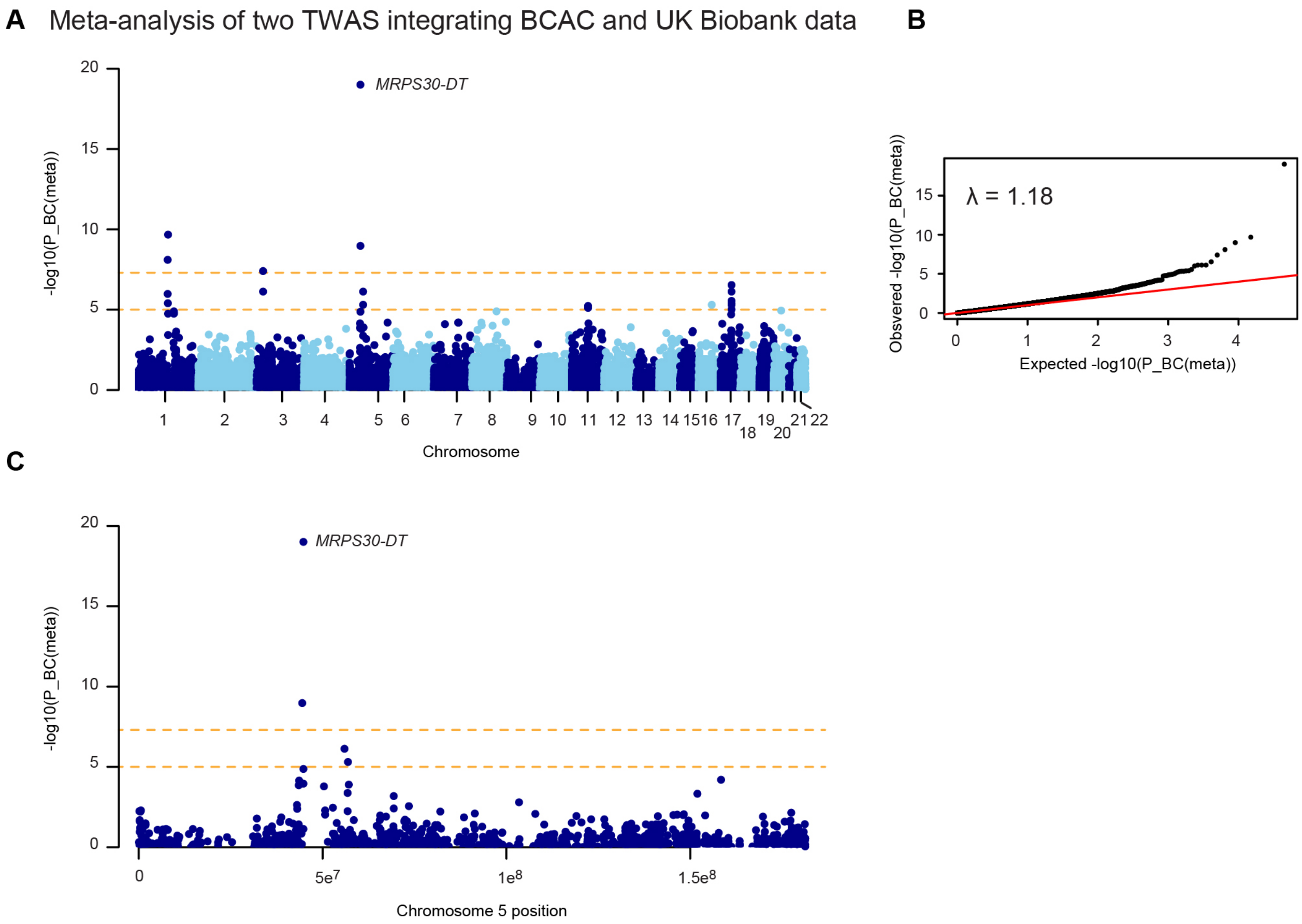


Figure S3. Meta-analysis of two TWAS integrating BCAC and UK Biobank datasets.

(A) Manhattan plot of gene-level TWAS association results from a meta-analysis combining BCAC (S-PrediXcan using GWAS summary statistics) and UK Biobank (PrediXcan) analyses. The x-axis shows autosomal chromosomes (1–22), and the y-axis shows −log10(P_BC(meta)). Each point represents a tested gene (including non-coding transcripts where applicable); the top association (MRPS30-DT) is annotated. Dashed orange lines indicate the significance thresholds ($P = 1 \times 10^{-5}$ and $P = 1 \times 10^{-8}$).

(B) Quantile–quantile (Q–Q) plot of observed versus expected −log10(P_BC(meta)) values for the meta-analysis TWAS results. The genomic inflation factor (λ) is shown, and the diagonal line indicates the null expectation.

(C) Regional TWAS association plot across chromosome 5, showing −log10(P_BC(meta)) values by genomic position; dashed orange lines indicate the same thresholds as in (A).

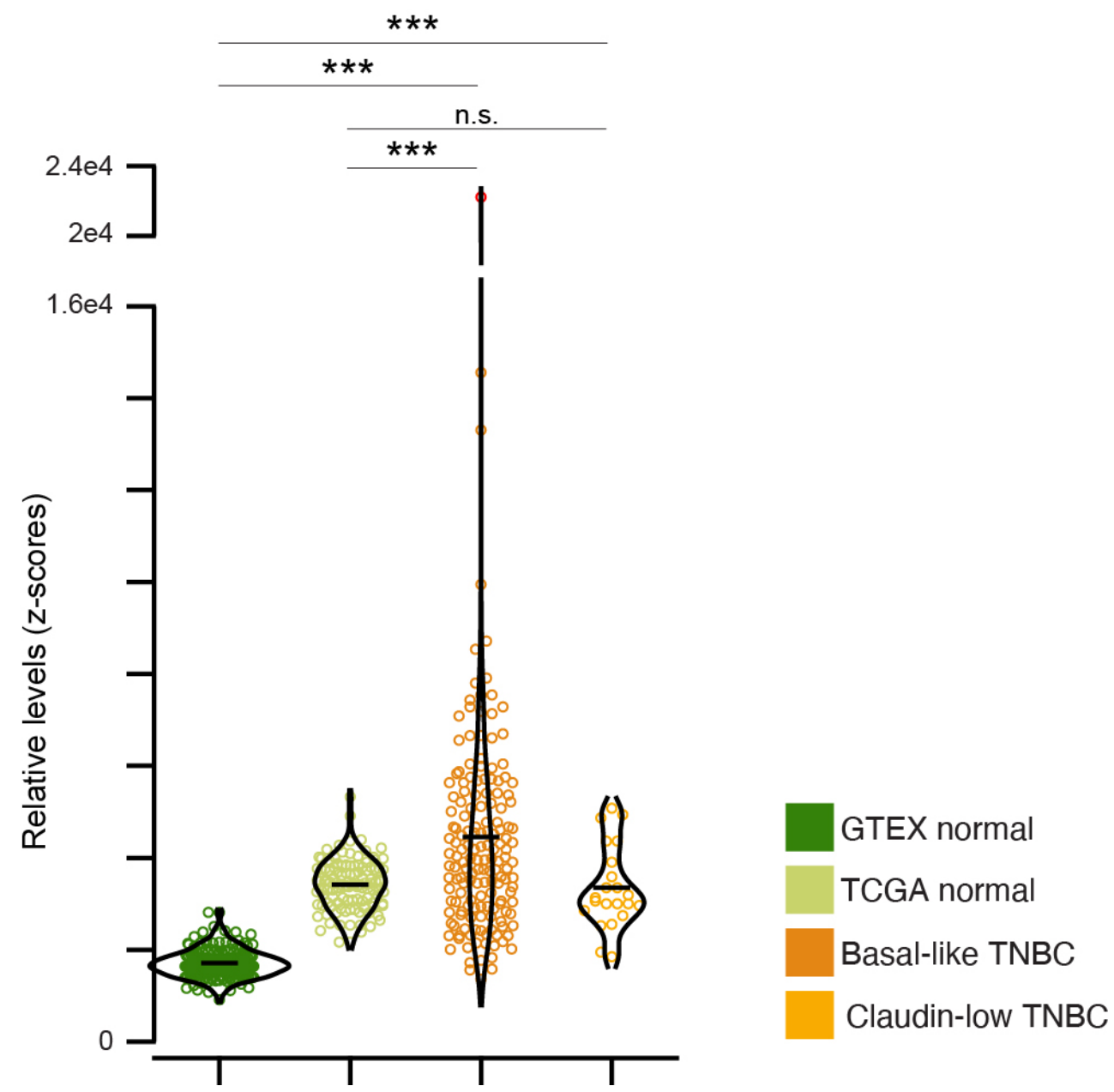


Figure S4. Subtype-dependent upregulation of SLC35E1 in TCGA TNBC.

SLC35E1 mRNA expression was quantified in GTEx normal breast tissue (n = 179), TCGA solid normal breast tissue (TNBCN; n = 113), TCGA basal-like TNBC (n = 162), and TCGA claudin-low TNBC (n = 22). Horizontal black bars indicate the mean expression (GTEx: 1705.27; TNBCN: 3414.17; basal-like: 4446.57; claudin-low: 3345.56). Pairwise comparisons were performed using Welch's two-sided t-test: GTEx vs basal-like ($p = 7.65\times10^{-31}$), GTEx vs claudin-low ($p = 2.58\times10^{-8}$), TNBCN vs basal-like ($p = 4.13\times10^{-7}$), and TNBCN vs claudin-low ($p = 0.733$; n.s.). Significance is denoted as *** $p < 0.001$; n.s., not significant.

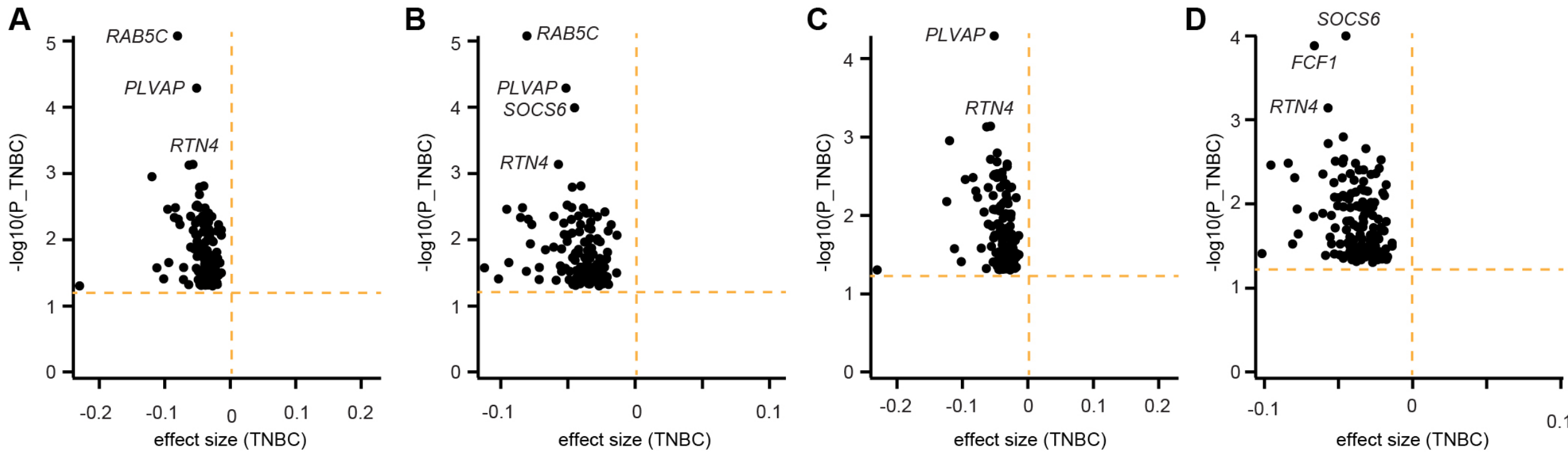


Figure S5. TWAS-prioritized genes negatively associated with TNBC risk and downregulated in TNBC subtypes.

(A–D) Scatter plots show genes that are negatively associated with TNBC risk in TWAS and are also downregulated in each TNBC subtype relative to the corresponding normal tissue. Plots display the relationship between the TWAS effect size (TNBC) (x-axis) and statistical significance (−log10(P_TNBC); y-axis). The vertical dashed orange line marks effect size = 0, and the horizontal dashed orange line denotes nominal significance (P = 0.05). The numbers of genes included are: GTEx normal vs basal-like TNBC (n = 131) (A), TCGA normal vs basal-like TNBC (n = 128) (B), GTEx normal vs claudin-low TNBC (n = 141) (C), and TCGA normal vs claudin-low TNBC (n = 138) (D). Selected top TWAS-prioritized genes are annotated.

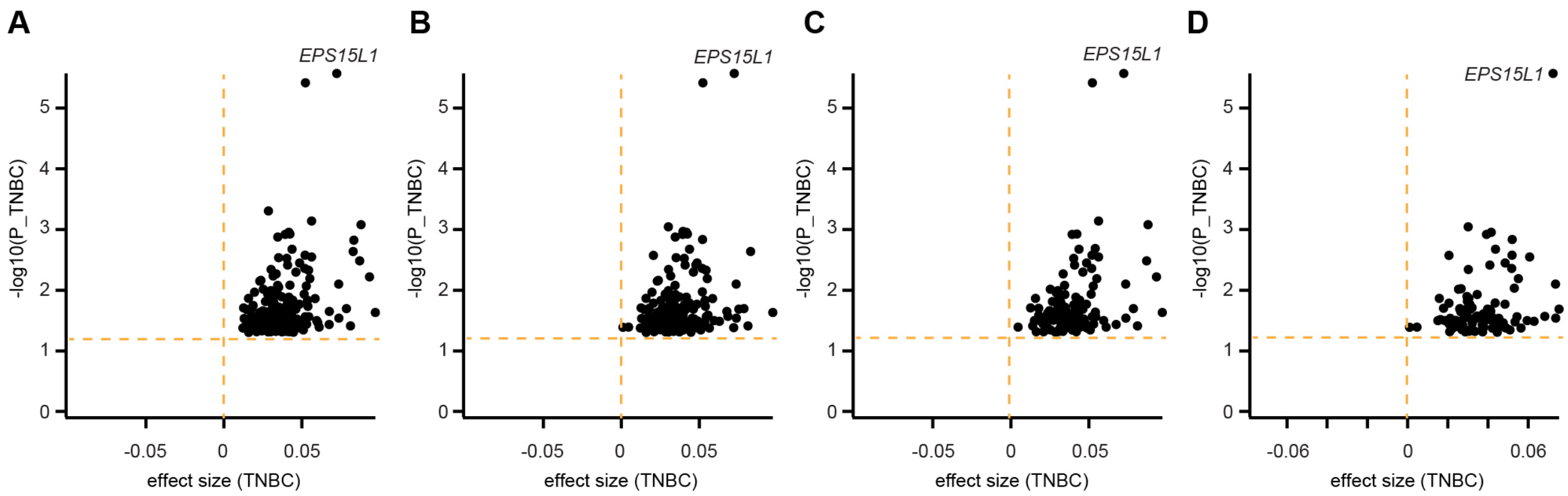


Figure S6. TWAS-prioritized genes positively associated with TNBC risk and upregulated in TNBC subtypes.

(A–D) Scatter plots show genes that are positively associated with TNBC risk in TWAS and are also upregulated in each TNBC subtype relative to the corresponding normal tissue. Plots display the relationship between the TWAS effect size (TNBC) (x-axis) and statistical significance (y-axis; −log10(P_TNBC)). The vertical dashed orange line marks effect size = 0, and the horizontal dashed orange line denotes nominal significance (P = 0.05). The numbers of genes included are: GTEx normal vs basal-like TNBC (n = 176) (A), TCGA normal vs basal-like TNBC (n = 161) (B), GTEx normal vs claudin-low TNBC (n = 132) (C), and TCGA normal vs claudin-low TNBC (n = 98) (D). EPS15L1 is annotated in each panel as a representative top TWAS-prioritized gene.

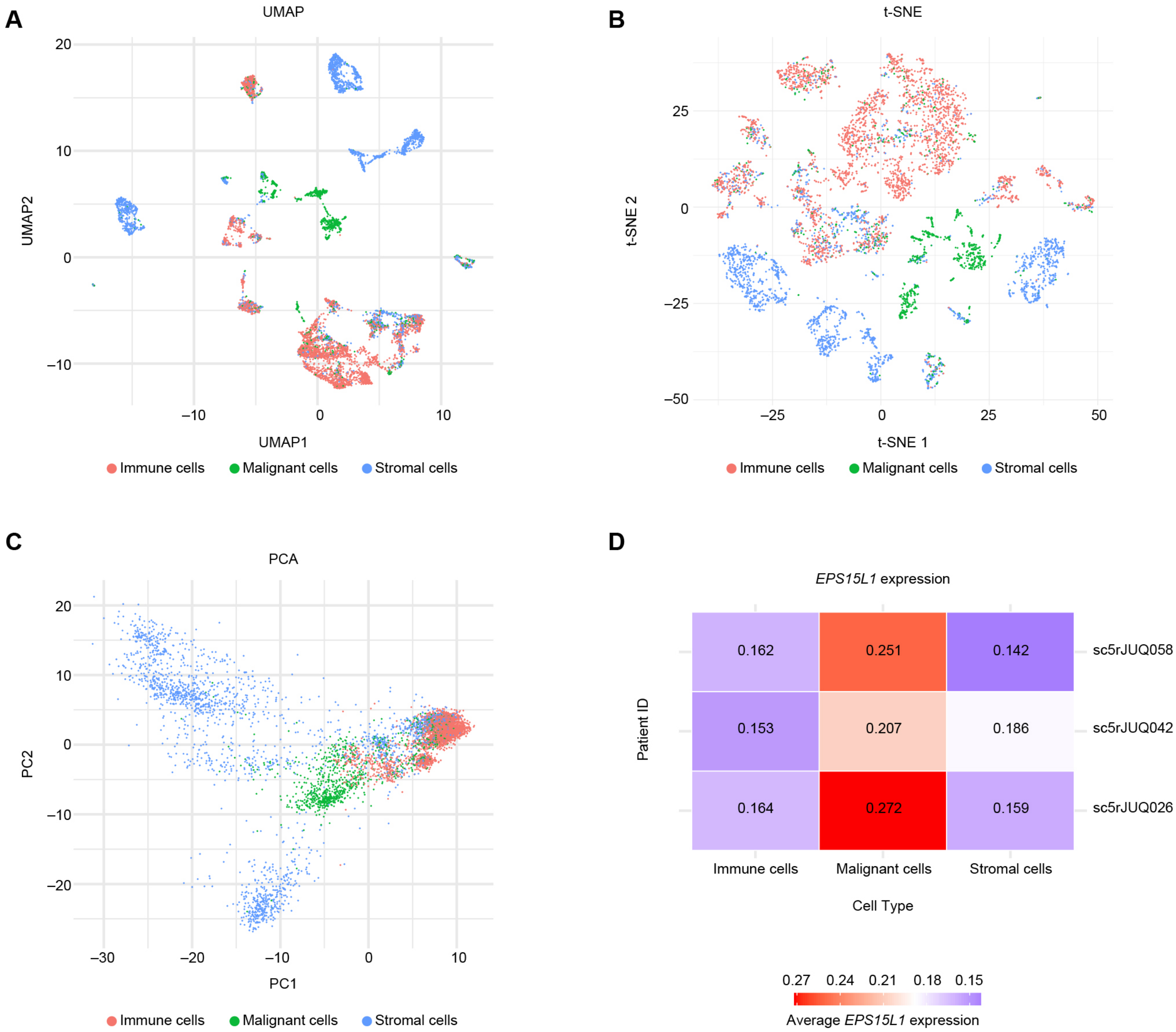


Figure S7. Single-cell analysis of the TNBC microenvironment reveals malignant-specific EPS15L1 expression.

(A) UMAP (Uniform Manifold Approximation and Projection) plot of single cells from three TNBC tumors (sc5rJUQ026, sc5rJUQ042, sc5rJUQ058), colored by annotated cell type (malignant-like epithelial, immune, stromal). The plot shows clear separation of the major cellular compartments.

(B) t-SNE (t-distributed Stochastic Neighbor Embedding) projection of the same dataset, confirming distinct clustering of malignant-like epithelial, immune, and stromal populations.

(C) PCA (Principal Component Analysis) plot colored by cell type, showing that the main principal components derived from highly variable genes effectively distinguish malignant-like epithelial cells from immune and stromal cells across patients.

(D) Heatmap displaying the average EPS15L1 expression in malignant-like epithelial, immune, and stromal compartments for each tumor, demonstrating that EPS15L1 is selectively upregulated in malignant-like epithelial clusters compared with immune and stromal populations.

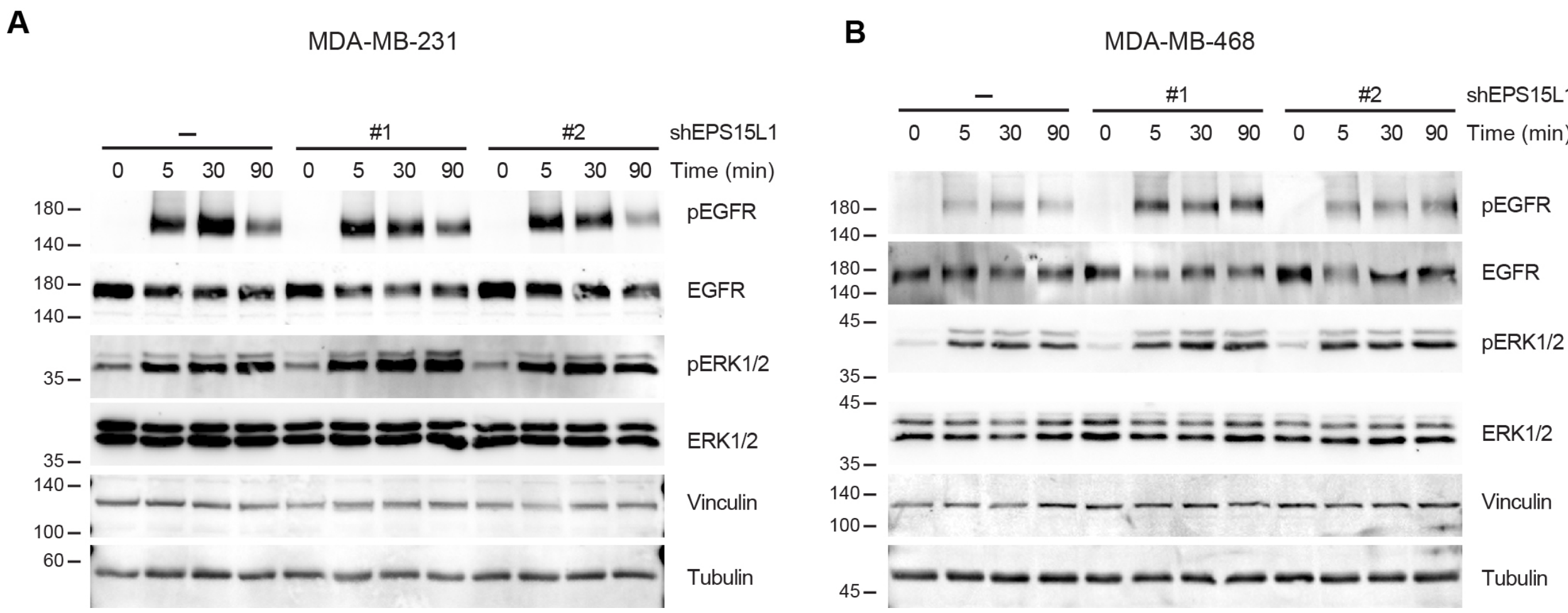


Figure S8. EPS15L1 knockdown does not alter acute EGFR-ERK signaling in TNBC cells.

(A) MDA-MB-231 and (B) MDA-MB-468 cells expressing doxycycline (DOX)-inducible shRNAs targeting Luciferase (shLuc control (–)) or EPS15L1 (shEPS15L1 #1 or #2) were serum-starved and stimulated with EGF (100 ng/mL) for 0, 5, 30, or 90 min. Whole-cell lysates were analyzed by immunoblotting for total EGFR, p-EGFR, total ERK1/2, and p-ERK1/2. Total EGFR abundance remained largely stable through 90 min in both cell lines. p-EGFR peaked at 5 min and declined thereafter, whereas p-ERK1/2 was induced at 5 min and sustained through 90 min. EPS15L1 knockdown did not alter EGFR levels or the kinetics of EGFR or ERK1/2 phosphorylation. Vinculin and Tubulin served as loading controls. P-values were calculated using Welch's two-sided t-test.

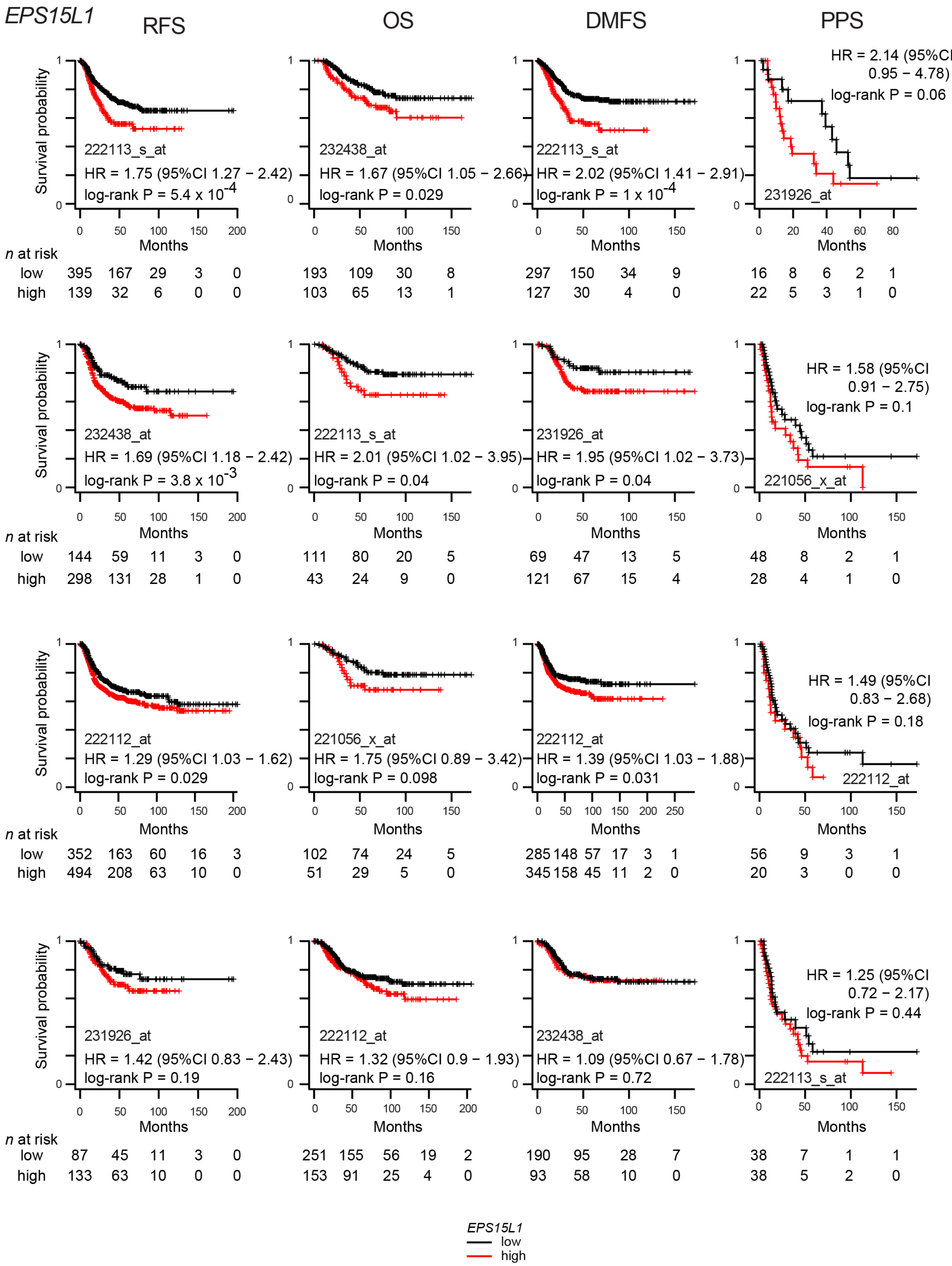


Figure S9. Kaplan–Meier survival analysis of EPS15L1 expression in TNBC.

Kaplan–Meier curves show survival outcomes for TNBC patients stratified by EPS15L1 mRNA expression (low, black; high, red) using the Kaplan–Meier Plotter database. Analyses were performed using the following Affymetrix probe sets for EPS15L1: 221056_x_at, 222112_at, 222113_s_at, 231926_at, and 232438_at. Relapse-free survival (RFS), overall survival (OS), distant metastasis-free survival (DMFS), and post-progression survival (PPS) are shown. For each plot, the hazard ratio (HR) with 95% confidence interval and log-rank P value are displayed, and numbers at risk are provided below the curves.

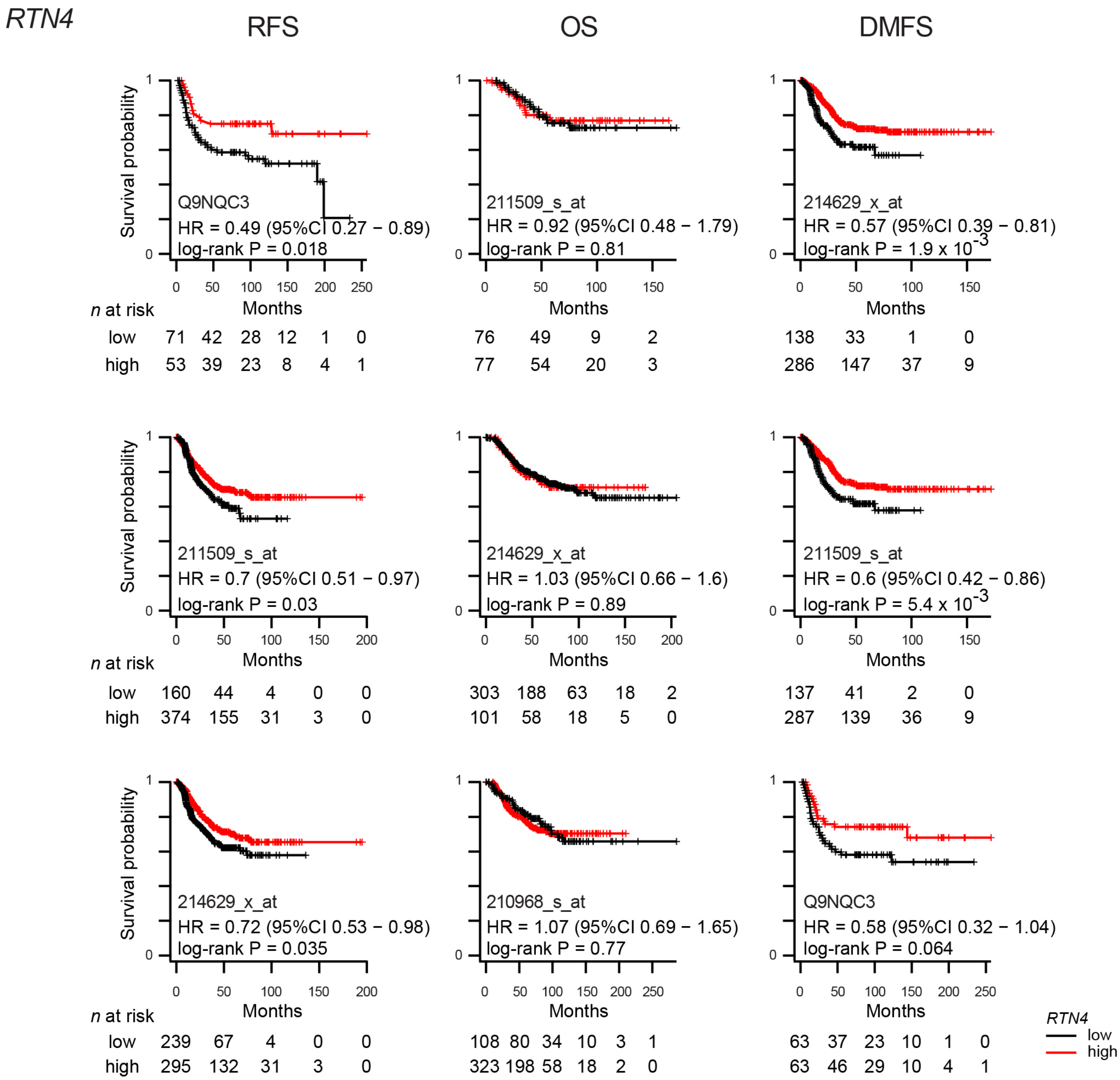


Figure S10. Kaplan–Meier survival analysis of RTN4 expression in TNBC.

Kaplan–Meier curves show survival outcomes for TNBC patients stratified by RTN4 mRNA expression (low, black; high, red) using the Kaplan–Meier Plotter database. Analyses were performed using the following Affymetrix probe sets for RTN4: 210968_s_at, 211509_s_at, and 214629_x_at; the UniProt accession for RTN4 is Q9NQC3. Relapse-free survival (RFS), overall survival (OS), and distant metastasis-free survival (DMFS) are shown. For each plot, the hazard

ratio (HR) with 95% confidence interval and the log-rank P value are reported, and numbers at risk are provided beneath the curves.

## Supplementary Data

Data S1. Comprehensive TWAS meta-analysis results (BCAC + UK Biobank) with TNBC association

This file provides gene-level TWAS summary statistics from the UK Biobank and BCAC, their meta-analysis, and TNBC-specific association results. Key fields include gene identifiers (Ensembl ID and gene symbol), cohort-specific effect sizes and P values, number of SNPs used in each model, meta-analysis Z-score and P value, concordance of effect directions across cohorts ("effect_product"), and TNBC effect size/P value with SNP count.

Data S2. EPS15L1 eQTL variant statistics and association results

This file lists eQTL variants used for EPS15L1-related analyses, including variant identifiers (rsID), genomic coordinates (chromosome, position), functional annotation (e.g., intronic/intergenic), locus/nearest-gene annotation, reference/effect alleles, and gene-expression model statistics (variant weight and P value). It also reports association estimates (beta, SE, P value) in TNBC (BCAC), overall breast cancer (BCAC), and overall breast cancer (UK Biobank).

Data S3. Pairwise linkage disequilibrium (LD) among EPS15L1 eQTL variants

This file contains a matrix of pairwise LD ($r^2$) values among the listed EPS15L1 eQTL variants (rsIDs shown as both row and column headers; diagonal = 1). These $r^2$ values support assessment of correlation/independence among the reported eQTL signals.

## Supplementary Table

Table S1. Genes exhibiting genome-wide significant negative associations with TNBC are implicated in tumor suppression.

| Gene name | Literature support (references in the main text) |
|---|---|
| *TACC1* | TACC1 is significantly correlated and downregulated in breast tumors (Conte *et al.*, 2003). |
| *TINAGL1* | Tinagl1 inhibits TNBC progression and metastasis by targeting both Integrin and EGFR signaling pathways (Shen *et al.*, 2019). |
| *L3MBTL1* | L3MBTL1, a polycomb tumor suppressor candidate in del(20q12) myeloid disorders, is essential for genome stability (Gurvich *et al.*, 2010). |
| *ECI2* | ECI2, a lipid-metabolism enzyme, may suppress colorectal cancer by inhibiting neutrophil extracellular traps (Chen *et al.*, 2024) |
| *CDKN1C* | ERK signaling suppresses the cell cycle inhibitor $p57^{Kip2}$, allowing proliferation to occur. The *CDKN1C* locus, which encodes $p57^{Kip2}$, is silenced in breast cancers (Worster *et al.*, 2012). |
| *PINK1* | PINK1-mediated suppression of EGFR signaling impedes lung tumorigenesis (Lin *et al.*, 2021). |
| *MIR22HG* | *MIR22HG* acts as a tumor suppressor in human primary lung tumors, downregulating cell cycle genes (Su *et al.*, 2018) |
| *BEND5* | BEND5-RBPJ interaction suppresses breast cancer growth and metastasis (Shi *et al.*, 2022). |
| *IRF2BPL* | Increased IRF2BPL expression, driven by FOXF2, inhibits gastric carcinogenesis (Higashimori *et al.*, 2018). |
| *RTN4* | RTN4 protein triggers apoptosis specifically in cancer cells, sparing normal cells (Li *et al.*, 2001). |
| *KLF11* | KLF11 expression levels predicted prognosis and chemotherapy response in sarcoma. Pharmacological KLF11 activation restored sensitivity to chemotherapy (Wang *et al.*, 2021). |
| *SOCS2-AS1* | lncRNA SOCS2-AS1 inhibits endometrial cancer progression (Jian *et al.*, 2021). |
| *RBPJ* | BEND5-RBPJ interaction suppresses breast cancer growth and metastasis (Shi *et al.*, 2022). |
| *TMEM170B* | TMEM170B is a novel breast tumor suppressor that targets the Wnt/β-catenin pathway (Li *et al.*, 2018). |
| *CHD1* | Loss of CHD1 drives resistance to AR-targeted therapy in prostate cancer (Zhang *et al.*, 2020). |

Table S2. Genes showing genome-wide significant positive associations with TNBC are implicated in EGFR endocytosis and signaling pathways.

| Gene name | Literature support (references in the main text) |
|---|---|
| *CBX3* | CBX3 promotes the malignant progression of glioblastoma multiforme by stabilizing EGFR expression (*Peng et al., 2022)*. |
| *SEC61B* | The translocon Sec61β, localized in the inner nuclear membrane, facilitates the transport of membrane-embedded EGFR to the nucleus (*Wang et al., 2010)*. |
| *AP1B1* | AP1B1, a clathrin adapter protein, regulates endosomal cargo trafficking to the plasma membrane (*Fields et al., 2007)*. |
| *ARSB* | ARSB is a potential therapeutic target in prostate cancer through its regulation of EGFR (*Bhattacharyya et al., 2018).* |
| *CHMP1A* | CHMP1A is essential for the function of the ESCRT-III complex, the major membrane remodeling machinery responsible for intra-lumenal vesicle formation on endosomal membranes (*Mercier et al., 2020).* |
| *EDEM2* | EDEM2 regulates the EGFR signaling pathway during *Drosophila* eye development (*Zettl et al., 2011)*. |
| *RELB* | RELB, an NF-κB transcription factor, functions as a co-activator of the EGFR-AP1 pathway (*Nottingham et al., 2014)*. |
| *RHOD* | RHOD is required for proper early endosome motility and distribution (*Murphy et al., 1996)*. |
| *SERPINE2* | SERPINE2 functions as a feedback regulator of EGFR signaling in breast cancer (*Tang et al., 2019).* |
| *PTPRO* | PTPRO counteracts ERBB2-driven breast cancer by promoting receptor dephosphorylation and internalization (*Dong et al., 2017*). |
| *RAB13* | RAB13 regulates EGFR levels and signaling by controlling EGFR trafficking and recycling (*Ioannou et al., 2016*). |
| *ABL2* | ABL2 tyrosine kinase modulates EGFR endocytosis and signaling, leading to enhanced EGFR activity and promotion of breast and lung cancer metastasis (*Hoj et al., 2020; Gu et al., 2016; Gil-Henn et al., 2013; Tanos et al., 2006*). |
| *EPS15L1* | EPS15L1 is responsible for driving residual EGFR-clathrin-mediated endocytosis in TNBC cells (*Pascolutti et al., 2019*). EPS15L1 mediates cholesterol-induced epithelial-to-mesenchymal transition through EGFR stabilization in prostate cancer cells (Jiang *et al.*, 2019). |
